# Bioreactor microbial ecosystems with differentiated methanogenic phenol biodegradation and competitive metabolic pathways unraveled with genome-resolved metagenomics

**DOI:** 10.1186/s13068-018-1136-6

**Published:** 2018-05-11

**Authors:** Feng Ju, Yubo Wang, Tong Zhang

**Affiliations:** 1 Environmental Biotechnology Lab, The University of Hong Kong SAR, Pokfulam Road, Hong Kong, China; 2 Institute of Advanced Technology, Westlake Institute for Advanced Study, Westlake University, Hangzhou, 310064 People’s Republic of China

**Keywords:** Genome-resolved metagenomics, Assembly and binning, Phenol degradation, Methanogenesis, Metabolic pathway, Process optimization

## Abstract

**Background:**

Methanogenic biodegradation of aromatic compounds depends on syntrophic metabolism. However, metabolic enzymes and pathways of uncultured microorganisms and their ecological interactions with methanogenic consortia are unknown because of their resistance to isolation and limited genomic information.

**Results:**

Genome-resolved metagenomics approaches were used to reconstruct and dissect 23 prokaryotic genomes from 37 and 20 °C methanogenic phenol-degrading reactors. Comparative genomic evidence suggests that temperature difference leads to the colonization of two distinct cooperative sub-communities that can respire sulfate/sulfite/sulfur or nitrate/nitrite compounds and compete for uptake of methanogenic substrates (e.g., acetate and hydrogen). This competition may differentiate methanogenesis. The uncultured *ε*-*Proteobacterium* G1, whose close relatives have broad ecological niches including the deep-sea vents, aquifers, sediment, limestone caves, spring, and anaerobic digesters, is implicated as a *Sulfurovum*-like facultative anaerobic diazotroph with metabolic versatility and remarkable environmental adaptability. We provide first genomic evidence for butyrate, alcohol, and carbohydrate utilization by a *Chloroflexi* T78 clade bacterium, and phenol carboxylation and assimilatory sulfite reduction in a *Cryptanaerobacter* bacterium.

**Conclusion:**

Genome-resolved metagenomics enriches our view on the differentiation of microbial community composition, metabolic pathways, and ecological interactions in temperature-differentiated methanogenic phenol-degrading bioreactors. These findings suggest optimization strategies for methanogenesis on phenol, such as temperature control, protection from light, feed desulfurization, and hydrogen sulfide removal from bioreactors. Moreover, decoding genome-borne properties (e.g., antibiotic, arsenic, and heavy metal resistance) of uncultured bacteria help to bring up alternative schemes to isolate them.

**Electronic supplementary material:**

The online version of this article (10.1186/s13068-018-1136-6) contains supplementary material, which is available to authorized users.

## Background

Methanogenesis of aromatic compounds is known to be affected by physical parameters [1–4], inhibitory chemicals (e.g., phenols and ammonium) [5], and substrate competitors that can outcompete methanogenic archaea [6]. Many efforts have been made to explore the microbial mechanisms behind physico-chemical or biological impacts by linking the community diversity and composition to biodegradation and/or methanogenesis rate under different environmental conditions [1, 3, 4, 7–9]. Methanogenic biodegradation of phenolic compounds, which are frequently detected as inhibitory intermediates in aromatic wastewater treatment bioreactors [1, 10], is implicated to reply on tightly coupled partnerships between syntrophic bacteria (e.g., *Syntrophorhabdaceae* and *Syntrophaceae*) and hydrogenotrophic methanogens. However, direct genetic and metabolic evidence for syntrophic phenol biodegradation and electron transfer in these methanogenic consortia is limited [8, 11]. Moreover, uncultured species of *ε*-*Proteobacteria*, *Chloroflexi* (e.g., T78 clade), *Aminicenantes* (candidate phylum OP8), *Synergistia*, *Bacteroidia*, and *Chlorobia* are widely detected in anaerobic phenol-degrading bioreactors [1, 3, 4]. Yet, the physiology and metabolism of these uncultured bacteria, their cooperative or competitive interactions with syntrophic bacteria and methanogens, and their impacts on the methanogenesis remain unknown mainly because of their resistance to isolation and the lack of their genomic information other than the 16S rRNA gene sequences.

Culture-independent genome reconstruction from multiple spatiotemporal environmental samples or their microbial enrichments by sequencing-based metagenomes and/or single-cell genomics is a powerful, and proven approach to access unprecedented genomic information of vast uncultured microbial dark matter (e.g., candidate phyla SAR406, OP3, OP11, WS1, BRC1, NKB19, OD1, TM7, and SBR1093) [12–17]. Further comparative genomics of uncultured microbes with their relatives of various degrees of phylogenetic relatedness, or with their functionally similar counterparts, reveals their phylogenetic distribution, functional diversity, evolutionary divergence, and the relationship between organismal phylogeny and functional traits, thus facilitating the understanding or prediction of their environmental niches and biological roles in natural and engineered biological ecosystems [15–18].

Here, we use genome-resolved metagenomics that integrates metagenome *de novo* assembly, binning, and comparative genomics to gain first genomic insights into the uncultured members in the previous phenol-degrading methanogenic bioreactors. Our study based on 16S rRNA gene sequence analysis reveals that temperature difference (37 °C vs. 20 °C) results in the bioreactor differentiation in the microbial compositions, phenol-degrading rate, and methanogenic efficiency [3], which, we hypothesize, is related to the differences in the biotic interactions and metabolic pathways besides their possible optimal growth temperature. The microbial inhibitory effect of phenol and its use as a sole organic carbon and energy source selectively enriched the methanogenic consortia and greatly simplified the community complexity, thus allowing successful reconstruction of high-coverage, high-quality genomic sequences from metagenomes with moderate and affordable sequencing depths as demonstrated in this study. Overall, the decoding of the phylogeny, physio-biochemical properties, and metabolic capacity of uncultured microorganisms reveals their metabolic potentials in methanogenic environments (e.g., anaerobic digesters) and lends novel insights into differentiated microbial syntrophic partnerships and competitive interactions during methanogenic phenol biodegradation.

## Methods

### Laboratory-scale bioreactors and selective enrichment

Microbial aggregates were propagated in laboratory-scale mesophilic (MP) and ambient (AP) methanogenic bioreactors semi-continuously fed with phenol as the sole organic carbon source, as reported previously [3]. Specially, for each gram of phenol feed, 119.0 mg MgSO_4_·7H_2_O and 16.7 mg NiSO_4_·7H_2_O were supplemented as nutrients, which is a major source of a small amount of sulfate to the bioreactors. The MP (37 °C) and AP (20 °C) bioreactor biomass, after 193-day enrichment, achieved distinct maximum methane-producing rate (200 and 283 mg-CH_4_/kg-VSS/day) and phenol-degrading rate (274.0 and 363.6 g-phenol/g-VSS/day) [3].

### Chemical analysis

Biogas composition (i.e., CH_4_, CO_2_, N_2_, and H_2_), and liquid volatile fatty acids (VFAs) and alcohols were detected using gas chromatograph, and liquid phenol and benzoate were measured using high-performance liquid chromatography (Shimadzu, Japan), as described previously [3]. Liquid nitrite (NO_2_^2−^) and nitrate (NO_3_^2−^) were monitored using ion chromatography with conductivity detector Anion/R column (Shimadzu, Japan).

### DNA extraction, library construction, and sequencing

Genomic DNA extracted with FastDNA SPIN Kit for Soil (MPBiomedicals, Illkirch, France) from the previous methanogenic phenol-degrading bioreactors [3] was re-sequenced using the Illumina Hiseq by applying a paired-end sequencing strategy (2 × 100 bp) with an library size of 800 bps (by ultrasonic shearing) at Beijing Genomics Institute (Shenzhen, China). Poor quality reads with any ambiguous nucleotide or with over 50% poor quality bases (i.e., quality score < 20) were trimmed. These generated clean data were co-analyzed with published metagenomes (2 × 100 bp) with an library insert size of 180 bps generated from the same DNA samples [3] to retrieve 23 prokaryotic genomes, following the bioinformatics analysis pipeline, as shown in Additional file 1: Figure S1.

### Metagenome assembly, binning, and genome evaluation

**Fig. 1:**
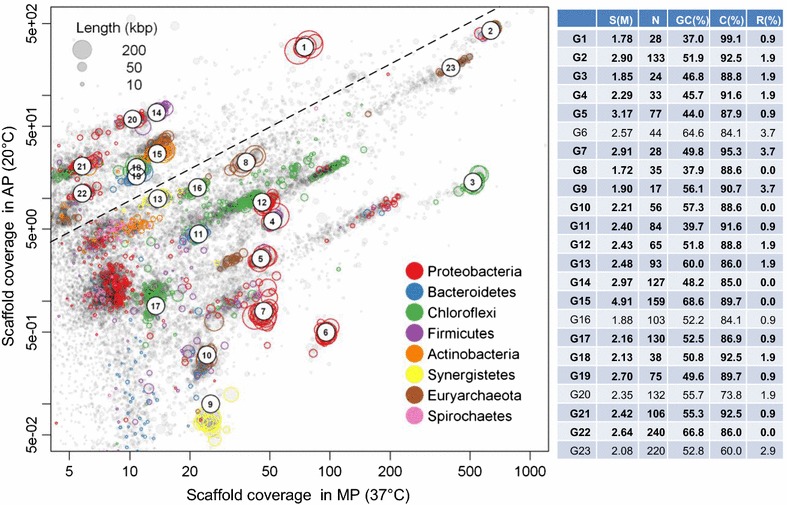
Differential coverage and composition binning of metagenome scaffolds from two methanogenic consortia. The consortia were selectively enriched using phenol under mesophilic (MP, 37 °C) and ambient (AP, 20 °C) conditions. Each circle represents a scaffold scaled by the square root of their length and colored according to their phylum-level taxonomic affiliation. Only scaffolds ≥ 1 kbp are depicted. Each cluster composed of circles with the identical color (labeled from G1 to G23) represents a putative genome. The dashed line depicts equal coverages in both metagenomes. *S* genome size (*M*), *N* number of scaffolds, *C* (%) and *R* (%): genome completeness and contamination (or redundancy), respectively, with genomes of over 85% completeness highlighted in bold

The clean reads of 180 and 800 bp library metagenomes of MP digester were *de novo* co-assembled in CLC Genomics Workbench v. 6.0.2 (CLC Bio; k-mer size: 51 bp) (Additional file 1: Table S1). Then, the scaffolds were grouped into putative genome bins using a composition-independent differential coverage binning pipeline [12]. In brief, clean reads from MP and AP metagenomes were mapped independently with Bowtie 2 (version 2.1.0) [19] to scaffolds of MP metagenomes (≥ 95% identity over full length). The scaffolds were then visualized in an *R* plot (with scaffold coverages, GC content, and taxonomic affiliations) and grouped into genome bins based on their two-dimensional coverages in MP and AP metagenomes (Fig. 1). After that, each resulting genome bin was purified by principal component analysis (PCA) based on tetranucleotide frequencies (TNFs) of the scaffolds to separate different species with similar coverage profiles. Finally, paired-end (PE) tracking was conducted to retrieve multiple-copy genes (e.g., 16S rRNA) that were not initially included in the coverage-defined bins.

### Genome abundance, completeness, and size estimation

The abundance of reconstructed genomes was calculated as the number of metagenomic reads mapped to each curated genome divided by the total number of reads in the metagenome using Bowtie 2 with default parameters. In total, 107 essential single-copy marker genes (ESCGs) and 35 conserved clusters of orthologous groups (COG) markers were used for estimates of completeness and potential contamination of 23 bacterial or archaeal genomes constructed from assemblies of MP and AP metagenomes. The first method is based on Hidden Markov Models (using hmmsearch with–cut_tc and–notextw) of 107 ESCGs (Additional file 1: Table S2) conserved in 95% of all bacteria [20], which can also be used to indicate the degree of potential contamination, because these genes are usually present in single copy. In the second method, a set of 35 orthologous group markers were used to evaluate completeness and contamination of archaeal genomes (Additional file 1: Table S2), as described before [21].

### Phylogenetic analysis of reconstructed genomes

Full-length or nearly-full-length 16S rRNA gene sequences were reconstructed from combined MP and AP metagenomes with EMIRGE [22] by running the program against reference database SILVA SSU 119 [23] clustered at 97% identity for 80 iterations. Reconstructed 16S rRNA gene sequences (> 1200 bp) post-chimera checking using ChimeraSlayer were annotated by BLASTN search against both SILVA SSU 115 and GreenGenes (2013–5 release) databases at an *e* value of 1*e*−20. The relative abundance of each 16S rRNA gene sequences was calculated by EMIRGE based on the length-normalized read number of mapped reads. The presence of rRNA gene sequences within each curated genome was identified using online RNAmmer 1.2 Server with default parameters [24], and then, the identified 16S rRNA gene sequence was further annotated by BLASTN search against SILVA SSU 115 and GreenGenes (2013–5 release) databases at an *e* value of 1*e*−20 [23, 25].

For phylogenetic analysis, 16S rRNA gene sequences *de novo* assembled using CLC assembler or reconstructed using EMIRGE were used for phylogenetic comparison between the curated genomes and reference sequences in the Silva SSU database. A maximum likelihood 16S rRNA gene phylogenetic tree was constructed with MEGA-5 [26] using 16S rRNA gene sequences aligned with MUSCLE and Jukes–Cantor model of nucleotide substitution and 100 bootstraps [27].

### Functional annotation and metabolic pathway analysis

The 23 curated genomes were annotated and deposited in IMG Data Management and Analysis Systems at the Joint Genome Institute (JGI; see Additional file 1: Table S3 for their genome IDs) [28]. The protein-coding genes, KEGG metabolic pathways, and clustered regularly interspaced short palindromic repeats (CRISPRs) regions were then predicted or annotated. The similarity in the KEGG metabolic capacity between a curated genome and its sequenced closest relatives (with complete genomes) was compared in PCA biplots using PAST3 [29]. To confirm gene annotation, protein-coding genes of each genome were downloaded from the JGI-IMG database and further re-annotated in a BLASTP search against the NCBI Reference Sequence Database (Nov, 2014) [30] and the UniRef90 Database (Jan, 2015) [31] at a maximum *e* value of 1*e*−5.

## Results and discussion

### Metagenome-assembled genomes

The genomic DNA from two phenol-degrading methanogenic bioreactors (operated for 193 days at mesophilic (37 °C, MP) and ambient temperatures (20 °C, AP) were sequenced, yielding 11.0 and 8.6 Gbp of clean paired-end reads (100 bps in length), respectively (Additional file 1: Table S1). The two MP data sets with library insert sizes of 180 and 800 bps were co-assembled into a total of 172 Mbp scaffolds with lengths ranging from 1 to 423 Kbp. Independent mapping of the raw reads of MP and AP samples to MP scaffolds and the visualization of coverage profiles in a two-dimensional coverage plot (Fig. 1) displays differential coverage of scaffolds, indicating that the identical microbial populations occurred with different abundances in the MP and AP bioreactors, which is also reflected by their different bacterial community composition revealed by read-based 16S rRNA gene analysis [3].

Grouping all scaffolds by their coverages clustered the scaffolds into 23 putative genome bins (labeled from G1 to G23, Fig. 1). These bins were further discriminated using a PCA of TNFs to remove potential contamination from other species in the identical coverage-defined bin, yielding refined genomes with sizes between 1.78 and 4.91 Mbp, GC content from 37.0 to 68.6%, and estimated genome completeness over 85% for most genomes (Fig. 1).

### Phylogenetic analysis reveals the novelty of the reconstructed genomes

**Fig. 2:**
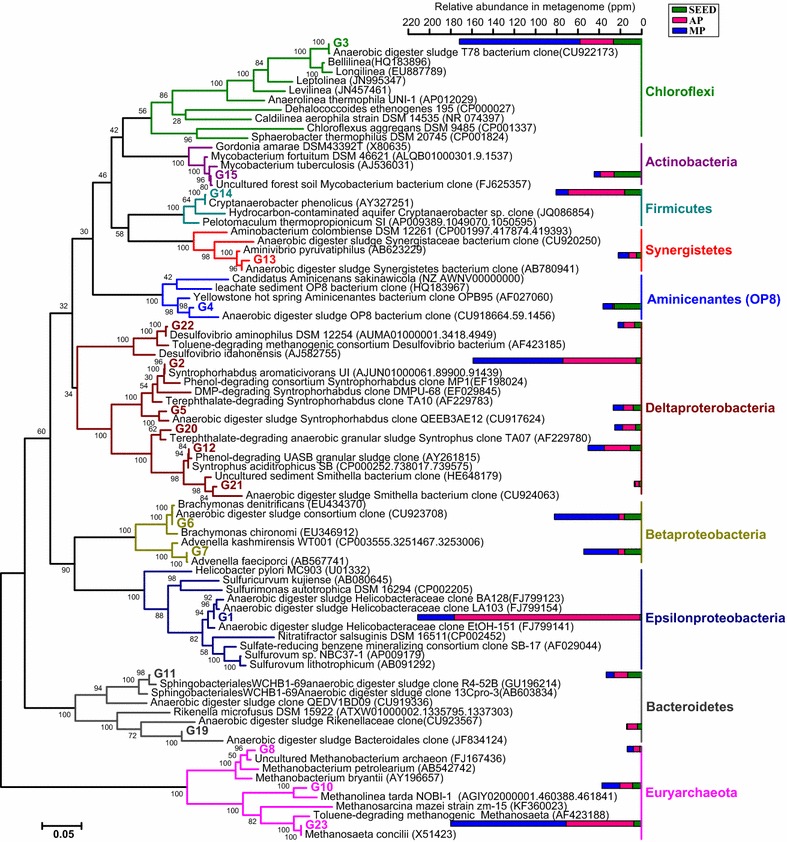
Maximum-likelihood 16S rRNA gene phylogenetic tree showing the placement of reconstructed genomes. The 16S rRNA genes of 4 out of total 23 genomes, namely, G9, G16, G17 and G18, are not successfully retrieved. The closest representative to each sequence is shown in parentheses (Accession Number). The accumulative bar chart indicates the relative abundance of each 16S rRNA gene sequence in the metagenomes of SEED, AP (20 °C) and MP (37 °C) sludge

Taxonomic assignment of full-length or near-full-length 16S rRNA gene sequences of the 19 curated genomes suggests that they originate from a wide range of phylogenetic distances (Fig. 2), including 16 members from 11 bacterial orders and 3 methanogens from three archaeal orders. Four genomes without near-complete (> 1000 bp) 16S rRNA genes, i.e., G9, G16, G17, and G18, were assigned according to the lowest common ancestors (LCAs) of their genomic essential single-copy marker genes (ESCGs), namely, to the families *Synergistaceae* (G9, 100% LCAs) and *Anaerolineaceae* (G16, G17, and G18 with 92, 85, and 86% LCAs).

The phylogenic analysis demonstrates that most of the reconstructed genomes belong to uncultured microorganisms previously known only by their 16S rRNA genes. The genome sequences were obtained for an uncultured *ε*-proteobacterium dominant in the AP (20 °C) reactor (G1, Additional file 1: Figure S2a, Table S3), a *Chloroflexi* T78 clade representative abundant in the MP (37 °C) reactor (G3, Additional file 1: Figure S2b), and less abundant uncultured microbes with different degrees of genetic novelty, including members of one unclassified OP8 class (G4, Additional file 1: Figure S2c), two unclassified *Bacteroidetes* families [WCHB1-69 (G11) and PL-11B8 wastewater-sludge group (G19)], two unclassified *Synergistaceae* genera (G13), two *Syntrophobacterales* genera (G20, G21), two euryarchaeotal genera [*Methanobacterium* (G8) and *Methanolinea* (G10)], and one *Mycobacterium* species (G15) (Fig. 2; Additional file 1: Table S3). Moreover, the 16S rRNA genes of G6 (1517 bp, 99.2%), G7 (1534 bp, 99.5%), and G14 (1526 bp, 99.9%) are nearly identical to *Brachymonas denitrificans* [32], *Advenella faeciporci* [33], and *Cryptanaerobacter phenolicus* [34], which are cultured species with isolates but still without a complete reference genome. The draft genomes obtained provide genomic insights into metabolic pathways of these bacterial populations in methanogenic environments, as discussed below.

### G1: an uncultured versatile *ε*-proteobacterial S/H_2_/N-metabolizer with remarkable environmental adaptability

Uncultured *ε*-Proteobacterium G1 is highly enriched in AP (20 °C) digester (Figs. 2; 6b). This microorganism and its uncultured relatives, i.e., 16S rRNA gene clones from methanogenic digesters [4, 35] or benzene-degrading enrichments [36–38], are embedded in the cluster of deep-sea hydrothermal vent sulfur-oxidizing chemolithoautotrophic *ε*-*Proteobacteria* (i.e., *Sulfurovum*, *Nitratifractor*, and *Sulfurimonas*) and most resembles *Sulfurovum* (Fig. 2). This phylogenetic relatedness is also evident in a PCA biplot depicting the similarities in KEGG metabolic profiles for all sequenced *ε*-Proteobacteria, in which the *Sulfurovum*-like G1 is clustered with hydrothermal vent strains (E90 and E98), whereas clearly segregated from the pathogenic *Helicobacter* (E31–E38) and *Campylobacter* species (E8–E30) (Additional file 1: Figure S3). However, G1 is probably a novel *ε*-*Proteobacteria* sublineage, because it shows only 93.2–94.4% 16S rRNA gene similarities to *Sulfurovum* spp. (i.e., NBC37-1, *S. lithotrophicum*, and recently nominated *S. aggregans*) and has a much smaller genome (1.78 Mb; > 99% completeness) than NBC37-1 (2.56 Mb). Genome-based physiological predictions demonstrate that G1 shares many genetic commonalities with *Sulfurovum* sp. NBC37-1 and other S/H_2_-oxidizing metabolizers, including *Nitratifractor*, *Sulfuricurvum*, *Nitratiruptor*, *Arcobacter,* and *Sulfuromonas* (Additional file 1: Figure S2a, Table S4).

#### Sulfur metabolism

**Fig. 3:**
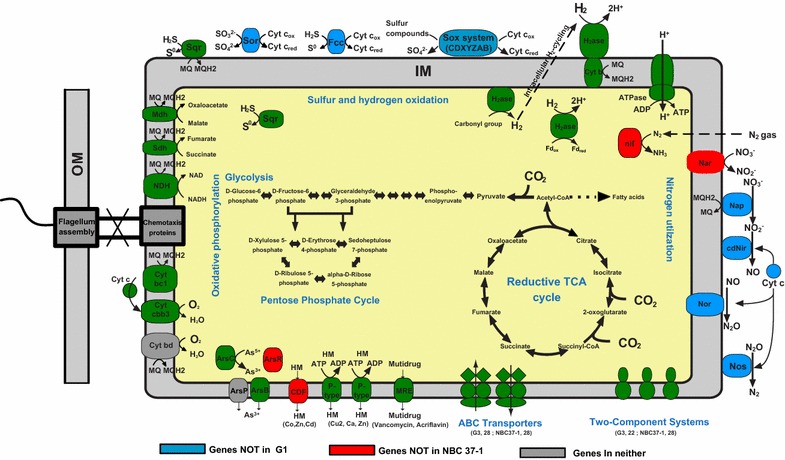
Central metabolism and solute transport in methanogenic digester *ε*-Proteobacterium G1. The closest cultured relative of G1, i.e., deep-sea vent *Sulfurovum* sp. NBC37-1, was used for comparison. Metabolic pathways for which enzymes are not encoded in either G1 or NBC37-1 but in other deep-sea vent *ε*-*Proteobacteria* are filled in grey. *IM* inner membrane, *OM* outer membrane, *Fd* ferredoxin, *Cyt* cytochrome, *H*_*2*_*ase* hydrogenase, *Sqr* sulfide–quinone oxidoreductases, *Fcc* flavocytochrome c sulfide dehydrogenase, *Nif* nitrogen-fixing proteins, *Nar* membrane-bound nitrate reductase, *Nap* periplasmic nitrate reductase, *cdNir* cytochrome cd1nitrite reductase, *Nor* nitric oxide reductase, *Nos* nitrous oxide reductase, *Mdh* malate dehydrogenase, *Sdh* succinate dehydrogenase (The figure was modified from Nakagawa et al. [40])

G1, like its autotrophic relatives (e.g., NBC37-1) [39–41], encodes all of the genes involved in the reductive tricarboxylic acid cycle (rTCA) for carbon (CO_2_) fixation and gene clusters for oxidative phosphorylation, hydrogen utilization (described below), polysulfide respiration, sulfide oxidation, and membrane-bound respiratory nitrate reduction (Fig. 3), providing the genetic basis for their versatile respiration. G1 has cytoplasmic and periplasmic sulfide:quinone oxidoreductases (Sqr) that can catalyze sulfide (HS^−^) oxidation to elemental sulfur (S^0^), a process perceived as contributing to filamentous sulfur formation in hydrothermal vents [40]. Moreover, G1 encodes genes involved in sulfur metabolism and homologous to those of NBC37-1, including (I) a polysulfide reductase cassette (PsrABC) that catalyzes polysulfide respiration coupled to hydrogen oxidation, (II) a sulfate adenylyltransferase (Sat) that catalyzes adenosine phosphosulfate oxidation to sulfate, and (III) a thiosulfate sulfurtransferase that catalyzes thiosulfate oxidation to sulfite. These genes indicate the similar capacity of the organism in utilizing sulfur compounds as both electron donors and acceptors (Additional file 1: Table S7-G1).

However, unlike NBC37-1, G1 has no sulfur-compound oxidation (Sox) system, which is typical in all other isolated or sequenced S/H_2_-oxidizing *ε*-proteobacterial chemoautotrophs except for *Sulfurospirillum deleyianum*, *Nautilia profundicola*, and *Thiovulum* sp. (Additional file 1: Table S4). Intriguingly, nitrogen fixation genes are encoded by both Sox-lacking G1 and *N. profundicola* with compact genomes (1.78 and 1.68 Mb, Additional file 1: Table S4-Yellow) and SoxCDYZ-lacking *Sulfuricurvum* and *Nitratifractor* (Additional file 1: Table S4-Green), whereas such genes are absent from those SoxCDYZ-carrying species of *Sulfurovum*, *Sulfurimonas* and *Arcobacter* (Additional file 1: Table S4-Purple). Combined, G1 might be a key player in driving sulfur cycling in the methanogenic reactors.

#### Hydrogen utilization as an energy source

Hydrogen-oxidizing sulfur respiration pathways using a hydrogenase and Psr have been noted in the energy metabolism of characterized deep-sea vent sulfur-metabolizing *ε*-*Proteobacteria* [42]. G1 also encodes four Ni–Fe hydrogenase subunits (2597532332-35, including two H_2_-uptake type and two H_2_-sensing type) and three Psr subunits ABC (2597532414-16) most similar to those in strain NBC37-1. The presence of these genes suggests the capacity to oxidizing hydrogen when using oxidized sulfur compounds as an electron acceptor. The presence of gene cassettes coding respiratory nitrate reductases (2597531175-77) and aerobic cbb3-type cytochrome c oxidase (2597531014-16) indicates that this bacterium is likely also capable of hydrogen oxidation by respiring nitrate or oxygen (Fig. 3).

Moreover, G1 contains complete H_2_-uptake hydrogenase clusters (e.g., HydABCD, HyaCD and HypFBCDEA, Additional file 1: Table S7-G1) which are well conserved in *ε*-*Proteobacteria* [40, 43]. By contrast, this bacterium, similar to the pathogenic *Helicobacter* species, lacks H_2_-evolving type of Ni–Fe hydrogenases typical in deep-sea vent *ε*-proteobacterial genomes (e.g., strains NBC37-1 and SB155-2) [40]. This type of hydrogenase is associated with the hydrogen release in formate (via formate dehydrogenase H) or carbon monoxide oxidation, or energy conservation during methanogenesis [44]. In addition, the presence of H_2_-sensing Ni–Fe hydrogenases in G1 (methanogenic digesters) and deep-sea vents strains, including NBC37-1 and SB155-2, is likely necessitated by the relative low concentrations of H_2_ in a methanogenic digester (typically < 0.1 μM [45]) or deep-sea vents (< 10 μM [40]). In summary, G1 is probably a substrate competitor of hydrogenotrophic methanogens (i.e., *Methanobacterium* and *Methanolinea*) in both methanogenic reactors, considering its full metabolic capacity in H_2_-oxidizing sulfur-compound respiration.

#### Environmental adaptation and ecological niches

G1 encodes plentiful enzymes to support microaerobic growth and cope with oxygen or oxidative stress (Additional file 1: Table S7-G1), including enzyme complexes I–V and cbb3-type cytochrome c oxidase that carries out a complete oxidative phosphorylation pathway (Fig. 3), antioxidant enzymes including alkyl hydroperoxide reductases and peroxidases, and iron cofactored superoxide dismutase. Moreover, like strain NBC37-1, G1 contains a large number of two-component regulatory system genes to sense and respond to changes in environmental cues, as well as a variety of transport enzyme systems to flexibly respond to environmental minerals or multidrugs, including (I) detoxification mechanisms of heavy metals including copper, cadmium, zinc, arsenate, and (II) resistance enzyme systems of acriflavin and vancomycin (Fig. 3 and Additional file 1: Table S7-G1). G1 possesses at least four couples of coding genes of chromosomal toxin–antitoxin (TA) systems (e.g., RelE–YefM, CopG–RelE, and YefM–YoeB). The exact roles of TA systems in cells are unclear, but their prevalence in bacterial genomes could be associated with stress resistance, population growth regulation, biofilm formation, or even niche-specific colonization [46–48]. In addition, G1 contains two regions of CRISPRs and CRISPR-associated protein-coding genes (IMG gene IDs: 2597532104-07; 2597531940-43), which may severe as defense systems against the invasion of exogenous genetic materials (e.g., phage infection).

Combined, our results suggest that *ε*-*Proteobacterium* G1 can be a facultative anaerobic metabolizer of hydrogen and sulfur compounds. These genetic potentials related to versatile metabolic capacities and remarkable environmental adaptability may provide solid foundations for the widespread distribution of G1-resembling 16S rRNA clones or populations (BLASTN similarity > 95%, bits core > 2300) in various artificial systems and environmental niches, such as benzene-degrading sulfate-reducing bioreactors [36–38], acetate-amended aquifers [49], antibiotic-receiving river sediments [50], sulfidic cave biofilms and springs [51–53], and limestone sinkholes [54]. Notably, recent protein stable isotope probing experiments with labeled acetate unveils some uncultured *Epsilonproteobacteria* as highly efficient dominant acetate scavengers in a sulfate-reducing microbial community mineralizing benzene [55]. Assume that G1 could also utilize acetate, it can compete with *Methanosaeta*, which are the dominant methanogens present in both bioreactors.

### T78 clade: phylogeny, occurrence, and metabolic potentials

Phylogenetic and taxonomic analysis of the full-length 16S rRNA gene sequences (1489 bp) of G3, which is markedly enriched in the MP reactor (Fig. 6b) shows that this T78 clade species is most closely related to members of *Anaerolineaceae*, including *Bellilinea* sp. clone De3218 (95.2%) and *Longilinea* sp. clone 48IIISN (95.0%), followed by *Leptolinea* BUT1_OTUB3 (88.9%), *Levilinea* clone SBYH_799 (88.9%), and *Anaerolinea thermophila* UNI-1 (87.2%) (Fig. 2; Additional file 1: Figure S2b). The T78 clade is a *Chloroflexi* cosmopolitan in freshwater lakes and springs [56, 57], sediments [58–60], and anaerobic digestion and biogas systems [61–63].

Genomic analysis shows that G3 encodes complete KEGG pathway enzymes for the beta oxidation of butyrate via crotonoyl-CoA to acetyl-CoA (Additional file 1: Figure S5a, Table S7-G3). G3 also has electron transfer flavoproteins (2600084386-87), NAD(P)-dependent iron-only hydrogenases (2600085382-85), and NAD(P) transhydrogenases (2600085557-60), which may be responsible for electron transfer and energy conservation via proton reduction (i.e., hydrogen production) coupled to proton translocation. Overall, the presence of these genes implicates that G3 is likely capable of butyrate oxidation. In addition, G3 has nearly a dozen ADH and AdDH enzymes for alcohol dehydrogenation and a complete list of enzyme-coding genes for KEGG glycolysis/gluconeogenesis and pentose phosphate pathways, suggesting that this T78 clade representative can metabolize alcohols and carbohydrates (Additional file 1: Table S7-G3).

### *Syntrophorhabdus*: key phenol-degrading syntrophs

Biodegradation mechanisms of aromatic compounds have been well documented in isolates of nitrate-reducing bacteria (NRB) [64, 65], SRB [66–68] and iron-reducing bacteria (IRB) [57]. Here, we reconstructed two near-complete genomes (G2 and G5) of genus *Syntrophorhabdus*, which is known to syntrophically degrade phenol under methanogenic conditions [69].

**Fig. 4:**
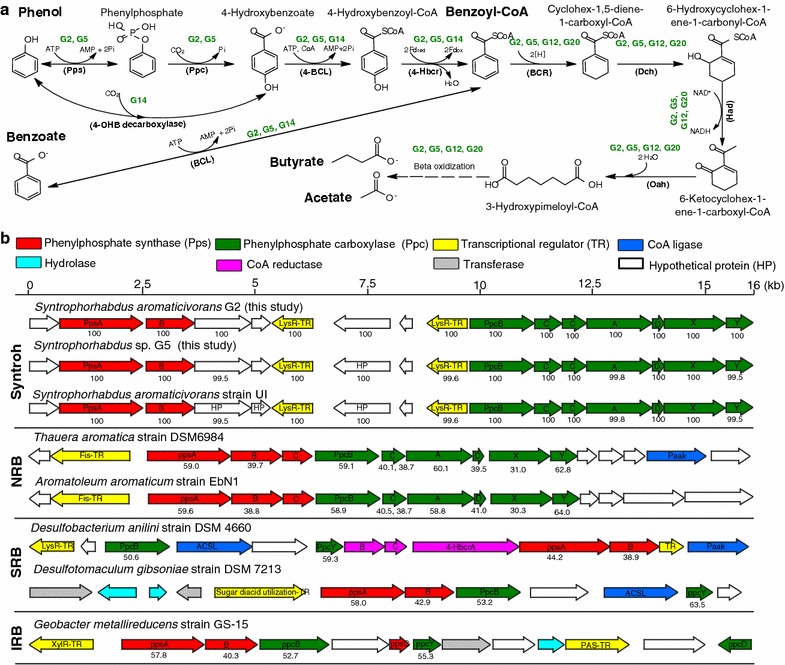
Anaerobic phenol and benzoate pathways (**a**) and the Pps-Ppc operons (**b**). **a** Phenol is anaerobically degraded to benzoyl-CoA, which is then degraded via ‘Dch-Had-Oah’ pathway and beta oxidation to butyrate and acetate. **b** The pathways can be conducted by anaerobes under methanogenic, nitrate-reducing, sulfate-reducing and iron-reducing conditions. The percentage under each enzyme-coding gene indicates its similarity (%) to the gene counterpart in *Syntrophorhabdus aromaticivorans* strain UI. *OHB* 4-hydroxybenzoate, *4-BCL* 4-hydroxybenzoate-CoA ligase, *4-Hbcr* 4-hydroxybenzoyl-CoA reductase, *BCL* Benzoate-CoA ligase, *BCR* Benzoyl-CoA reductase, *Dch* Cyclohexa-1,5-dienecarbonyl-CoA hydratase, *PaaK* putative phenylacetate-CoA ligase, *Had* 6-hydroxycylohex-1-en-1-carbonyl-CoA dehydrogenase, *PaaK* putative phenylacetate-CoA ligase, *Oah* 6-oxo-cyclohex-1-ene-carbonyl-CoA hydrolase, *PaaK* putative phenylacetate-CoA ligase, *ACSL* long-chain acyl-CoA synthetase

Phylogenetic distance of 16S rRNA gene, in silico DNA–DNA hybridization value (DDH), average amino acid identity (AAI), and average nucleotide identity (ANI) (Additional file 1: Table S5) congruously reveal that G5 is from a novel *Syntrophorhabdus* species (Fig. 2; Additional file 1: Table S5). A gene-by-gene manual comparison suggests this organism shares numerous gene cassettes with *S*. *aromaticivorans* (G2 and strain UI) for (I) the syntrophic biodegradation of phenol, 4-OHB, benzoyl-CoA (using non-ATP-dependent benzoyl-CoA reductase, Additional file 1: Table S7) and benzoate (Fig. 4a and Additional file 1: Figure S4) and (II) a novel reverse electron transport mechanism [11] in Rnf-lacking syntrophic metabolizers of aromatic compounds (Additional file 1: Text S1).

A further comparison of the phenylphosphate synthase and carboxylase gene clusters (Pps–Ppc operons) in *Syntrophorhabdus* with those encoded by NRB, SRB, and IRB reveals that they share essential gene subunits, i.e., PpsAB and PpcBY in the Pps–Ppc operons (Fig. 4b). The PpcX (UbiD-like) and PpcY (UbiX-like) subunits encoded in the downstream of Pps–Ppc operons of syntrophs and NRB were distantly homogenous to 4-hydroxybenzoate (4-OHB) decarboxylases (BLASTP similarity: 28 and 47%; bit score: 170 and 172), which are enzymes responsible for phenol decarboxylation to 4-OHB [70]. It has been demonstrated that PpcX was transcribed to directly carboxylate phenol to 4-OHB in iron-reducing archaea *Ferroglobus placidus* [71], while PpcY (originally referred as ORF8 in *T. aromatica*) was transcribed alongside PpsAB and PpcB genes in *Geobacter metallireducens* during anaerobic growth on phenol [72]. Based on these observations, it is speculated that PpcX and PpcY are putative 4-OHB decarboxylases responsible for a previously unrecognized phenol decarboxylation pathway in *Syntrophorhabdus*.

### *Cryptanaerobacter*: unrecognized genetic contents and metabolic pathways

**Fig. 5:**
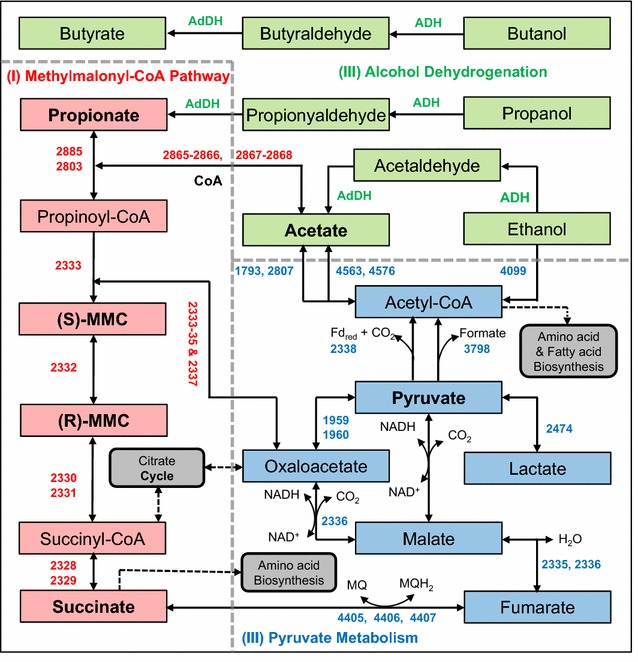
KEGG biodegradation pathways encoded in the genome of *Cryptanaerobacter* sp. G14. Each reaction is labeled with the abbreviated IMG gene IDs (e.g., ‘2600290001’ as ‘0001’) for the enzyme-coding genes. *ADH* alcohol dehydrogenases, *AdDH* aldehyde dehydrogenases, *MQ* menaquinone, *Fd* ferredoxin

The first and only isolate of *Cryptanaerobacter*, i.e., strain LR7.2, was described as an anaerobe that presumably utilized phenol or 4-OHB as an energy source and electron acceptor for growth in pure culture with essential complex supplements, seemingly converting these compounds into benzoate via unknown “unusual anaerobic respiration” [34]. However, the authors could neither identify electron donors and carbon sources, nor explain the stimulated growth of strain LR7.2 by sulfite. For more than a decade, studies have been investigating whether *Cryptanaerobacter*, like its *Pelotomaculum* relatives [73], syntrophically oxidizes organic substrates, or like its *Desulfitobacterium* relatives [74] performs sulfite/sulfonates reduction. In this study, we present genomic evidence that G14 performs (I) phenol/4OHB degradation and assimilatory sulfite reduction and (II) syntrophic propionate oxidation (Fig. 5) in methanogenic environments.

Gene-by-gene analysis of the first *Cryptanaerobacter* genome shows that unlike phenol-degrading *Syntrophorhabdus*, NRB, SRB, and IRB that utilize a phosphorylation–carboxylation pathway to convert phenol to 4-OHB, G14 encodes homologs of 4-OHB decarboxylase subunits BCD (2600292530-32, Additional file 1: Table S6) that convert phenol into 4-OHB via a carboxylation pathway (Fig. 4a). Moreover, G14 has the genes coding putative 4-hydroxybenzoate-CoA ligases (4-BCL, 2600292885), putative 4-hydroxybenzoyl-CoA reductase (4-HBCR, 2600294143-44), and benzoate-coa ligase (BCL, 2600294302) to further convert 4-hydroxybenzoate (4-OHB) to benzoate (Additional file 1: Table S6). Notably, G14 encodes an anaerobic sulfite reductase (asrABC; 2600293676-78), NADPH-dependent flavin oxidoreductases (e.g., 2600293674), and putative NADPH-dependent hemoprotein (2600293675), which are engaged in the NADPH-dependent assimilatory sulfite reduction (ASR) to sulfide via the following reaction:

$${\text{SO}}_{3}^{2 - } + 3{\text{NADPH}} + 3{\text{H}}^{ + } \leftrightarrow {\text{S}}^{2 - } + 3{\text{NADP}}^{ + } + 3{\text{H}}_{2} {\text{O}} .$$

Therefore, G14 may perform phenol decarboxylation and assimilatory sulfite reduction to derive energy for growth. These metabolic capacities of G14 make the previously-observed sulfite stimulated growth of *C. phenolicus* plausible [34]. Notably, G14 encodes three complete sets of ABC-type transport systems for sulfonate (Additional file 1: Table S6), a substrate utilizable by its sulfite-reducing *Desulfitobacterium* relatives as a terminal electron acceptor (TEA) for growth [74]. Nevertheless, to ascertain whether G14 assimilates sulfonate is impossible merely by reference-based genome annotation, because the enzymes responsible for sulfonate assimilation in *Firmicutes* remain unknown. Assuming that the ABC-type sulfonate transport system, sulfite reductases, and hypothetical proteins of G14 enable its uptake of sulfonate, phenol/4-OHB may also be degraded via sulfonate respiration. Future studies are needed to test whether sulfonate supports growth of *Cryptanaerobacter* species and which enzymes will be expressed by *Desulfitobacterium* isolates to utilize (aliphatic) sulfonate as TEA for growth with the release of sulfonate sulfur as sulfide.

On the other hand, G14 encodes a complete methylmalonyl-CoA (MMC) pathway for the syntrophic conversion of propionate into succinyl-CoA and succinate. These two compounds are then metabolized via oxaloacetate to pyruvate and eventually transformed to acetyl-CoA and acetate through a series of enzymatic reactions (Fig. 5 and Additional file 1: Table S6). The MMC pathway is a common mechanism for propionate oxidation in many mesophilic syntrophs, and the eight catabolic genes encoding the MMC pathway in G14 are most similar (66–89%, averaged 81%, Additional file 1: Table S6) to those of its closest cultured relative, *Pelotomaculum thermopropionicum* SI (Fig. 2, 93.8% 16S similarity; AAI: 53.2%), a syntrophic propionate-oxidizing thermophilic anaerobe [75]. Like strain SI, G14 encodes at least five alcohol dehydrogenases (ADH) and one acetaldehyde dehydrogenase (AdDH) for alcohol (e.g., propanol) oxidation (via aldehyde) to carboxylic acids (Fig. 5 and Additional file 1: Table S6), such as propionate. The propionate could be further oxidized by G14 in syntrophy (with a hydrogen-scavenging partner) using an ion-translocating ferredoxin oxidoreductase genes (IFO)-associated cassette (2600291645-53, Additional file 1: Table S6) that encodes a heterodisulfide reductase complex, hydrogenase subunits, and putative ion-translocating Fd:NADH oxidoreductase subunits for energy conservation, as is the case for many other Rnf-lacking syntrophic metabolizers [11]. Moreover, genome analyses reveal two iron-only hydrogenases and one Ni-Fe hydrogenase in G14 (Additional file 1: Table S6), presumably engaging in discharging reducing equivalents (i.e., electrons) after propionate oxidation via three reductive steps, namely, menaquinone reduction by a succinate dehydrogenase (2600294405-07), NAD^+^ reduction by a malate dehydrogenase (2600292336), and ferredoxin reduction by a pyruvate/ferredoxin oxidoreductase (2600292338) (Fig. 5). In addition, G14 has two gene clusters containing formate dehydrogenases. However, whether G14 can produce formate to exhaust reducing equivalents or pool electrons requires further investigation.

Combined, the genomic evidence reveals that *Cryptanaerobacter* sp. G14 may play dual flexible and significant roles in methanogenic environments, both as a sulfite-respiring, aromatic compound degrader (i.e., phenol and 4-OHB) and a syntrophy specialist that uptakes volatile fatty acids (e.g., propionate) and alcohols (e.g., propanol, ethanol) generated by upstream fermentative or acidogenic bacteria and syntrophically degrades them to acetate, hydrogen, and carbon dioxide. These compounds are then available as substrates for downstream methanogens.

### Nitrate/nitrite denitrifiers in 37 °C phenol-degrading consortia

KEGG pathway annotation of *Burkholderiales* genomes, i.e., G6 and G7, identifies gene cassettes and metabolic pathways (Additional file 1: Table S7-G6 and G7) related to (I) nitrate and/or nitrite denitrification (e.g., Additional file 1: Figure S5b for G6), (II) RnfABCDGE type electron transfer, (III) oxygen/oxidative tolerance, (IV) propionate biodegradation (via acryloyl-CoA pathway) (Additional file 1: Figure S5c-III for G6), and (V) metabolism of pyruvate and lactate. These genetic potentials are well supported by the fully validated capacities of their closest cultured isolates, namely, *B. denitrificans* and *A. faeciporci* (Fig. 2), to use these organic substrates as carbon and energy sources [32, 33].

In agreement with their oxygen/oxidative tolerance, G7 encodes two gene cassettes for oxic/anoxic biodegradation of 4-OHB (to pyruvate, Additional file 1: Figure S5d-I) and catechol (to acetyl-CoA using catABC and pcaDIJ operons, Additional file 1: Figure S5d-II and Table S7-G7), while G6 encodes four enzymes that catalyze oxic/anoxic oxidation of benzoate and phenol (to catechol; Additional file 1: Table S7-G6). Moreover, protein-coding genes related to amino acid metabolism are the most abundant in both G6 (169 KEGG orthology, i.e., KO) and G7 (201 KO), followed by those genes involved in utilization of carbohydrates (G6: 134 KO; G7: 158 KO). These genome-encoded metabolic potentials of G6 and G7 are well in agreement with the validated capacities of their closest relatives to utilize a variety of amino acids as energy and carbon sources for anaerobic growth [32, 33]. Notably, besides an acryloyl-CoA pathway, G6 also encodes all enzymes essential for propionate degradation via a MMC pathway (Additional file 1: Figure S5c-I) and a reductive carboxylation pathway (Additional file 1: Figure S5c-II and Table S7-G6).

### Sulfate and sulfite metabolizers in the 20 °C phenol-degrading consortia

**Fig. 6:**
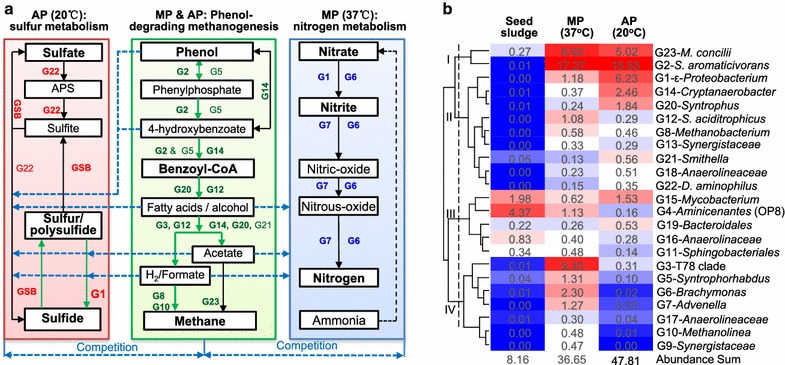
Substrate competition between methanogenic consortia and sulfur/nitrogen compounds metabolizers. **a** MP: 37 °C; AP: 20 °C. Green arrow lines implicate syntrophic phenol-degrading methanogenic pathway. Blue arrow lines indicate pathway competition for methanogenic substrates and other organic molecules. *APS* adenosine 5′-phosphosulfate, *GSB* green sulfur bacteria *Chlorobiaceae*. **b** Percent in heatmap cells denotes relative abundance (%) of each genome in a metagenome

Three genomes, namely, G15, G21, and G22, are more enriched in the AP reactor (Fig. 6b), display full genetic capacities to utilize sulfur compounds as electron acceptors.

G15 encodes complete enzymes (i.e., CysND, CysC, CysH, and sir) for assimilatory sulfate reduction via sulfite to sulfide (Additional file 1: Table S7-G15). Consistent with most of its well-understood *Mycobacterium* relatives [76, 77], this uncultured facultative anaerobic organism encodes biodegradation enzymes of xenobiotic polycyclic aromatic hydrocarbons (e.g., naphthalene, dichloropropene, and phenanthrene), carbohydrates (e.g., starch, sucrose, and glucose), and fatty acids (i.e., hexadecanoate, butyrate, and propionate).

*Smithella* sp. G21 encodes enzymes for syntrophic oxidation of propionate (i.e., MMC oxidation pathway and acryloyl-CoA pathway) and butyrate (via crotonoyl-CoA), an iron-only hydrogenase, formate dehydrogenases, alcohol dehydrogenases, dissimilatory sulfite reductases (DsrABD, 2603687864-66) and associated electron transfer proteins (DsrKJO), and polysulfide reductases (Psr) (Additional file 1: Table S7-G21), revealing its potential in VFAs and alcohol metabolism, hydrogen production, formate oxidation, and the uptake of sulfur compounds as electron acceptors.

G22 resembles *Desulfovibrio aminophilus* DSM 12254 (99.9% 16S similarity, Fig. 2; 70% DDH), an amino acid-degrading and sulfate-reducing bacterium (isolated from an anaerobic dairy wastewater lagoon) that also utilizes formate, H_2_/CO_2_, and ethanol as electron donors [78].

### Differentiated methanogenic phenol-degrading metabolic pathways

Tracking habitat origins of close relatives of uncultured microorganisms in our MP and AP digesters by their reconstructed 16S rRNA genes reveals that these microbes are widespread in methanogenic bioreactors or enrichments receiving wastewater-borne aromatic compounds and sulfate [1, 3, 4, 35–37]. The temperature difference between MP (37 °C) and AP (20 °C) bioreactors acclimates two divergent methanogenic communities that significantly differ in community composition (Figs. 1 and 6b), and methane-producing rate (200 vs. 283 CH_4_-COD/g-VSS/day) and phenol-degrading rate (274.0 and 363.6 g-phenol/g-VSS/day) [3]. Besides potential temperature dependence of biodegradation rate, genome-resolved evidence suggests that the higher methane production and faster phenol degradation at 37 than 20 °C are associated with different microbial syntrophic and competitive relationships (Fig. 6a) besides potential differences in enzymatic activities, as described below.

In both MP and AP reactors where effluent monitoring on Day 113, 130, 146, 151, and 167 suggests the presence of benzoate, acetic acid, ethanol, and butanol (Fig. 6a), phenol is syntrophically degraded to benzoyl-CoA by *Syntrophorhabdus* spp. (G2 and G5) in the presence of hydrogen-scavenging microorganisms, such as the hydrogenotrophic archaeal *Methanobacterium* (G8) and *Methanolinea* (G10). Phenol can be also converted to 4-OHB and benzoyl-CoA by *Cryptanaerobacter* sp. G14. Then, benzoyl-CoA is degraded by *Syntrophorhabdus* spp. and other syntrophs, such as *Syntrophus* spp., to fatty acids (e.g., butyrate/propionate/acetate) and/or alcohols (e.g., butanol/propanol/ethanol). These byproducts can be further selectively oxidized to methanogenic substrates (i.e., acetate, formate, or hydrogen) by syntrophs, including 37 °C-enriched T78 clade bacterium (G3) and *Syntrophus aciditrophicus* (G12), and/or 20 °C-enriched (G14), *Syntrophus* (G20) and *Smithella* (G21). After that, acetate is used by *M. concilii* (G23) for direct acetoclastic methanogenesis, whereas hydrogen and formate are probably utilized by hydrogenotrophic *Methanobacterium* (G8) and *Methanolinea* (G10) to reduce carbon dioxide to methane.

However, the temperature difference induces the great shift of community composition in the seed sludge (Fig. 6b) and the development of two distinct but cooperative sub-communities that, respectively, metabolize sulfate/sulfite/sulfur and nitrate/nitrite in AP and MP reactors, leading to potential substrate competition with methanogens and syntrophic bacteria (Fig. 6a). In the AP reactor, sulfate is converted to sulfide through dissimilatory reduction by SRB *Desulfovibrio* (e.g., G22) and assimilatory reduction by *Mycobacterium* spp. (G15). This accompanies competitive uptakes of methanogenic substrates with methanogens (i.e., hydrogen, formate, acetate) or of small organic substrates (e.g., ethanol or amino acids) with syntrophs. Sulfite can be assimilated by *Cryptanaerobacter* (G14) to stimulate its growth and 4-OHB transformation activity. Sulfide, a notorious and ubiquitous product in anaerobic digestion processes, is oxidized by phototrophic green sulfur bacteria (GSB) *Chlorobiaceae*, which occurred only in the AP reactor and accounted for 3.6 and 4.5% of the amplicon and metagenome 16S rRNA gene sequences, respectively [3], to regenerate sulfite and sulfate, yielding element sulfur as an intermediate product. The element sulfur and polysulfide are reducible by uncultured *ε*-proteobacterium G1 and *Smithella* spp. G21 with the oxidation of hydrogen and formate, respectively. Therefore, SRB (*Desulfovibrio*), sulfur-reducing bacteria (G1), and GSB (*Chlorobiaceae*) can form a cooperative metabolic network in which sulfur compounds are recycled and exchanged among the partners. This leads to continuous competitive depletion of methanogenic substrates, thus deteriorating methane production by methanogens.

In contrast, the co-occurrence of substrate competitors, i.e., nitrate/nitrite-denitrifying *Brachymonas* sp. G6, nitrate-reducing G1, and nitrite-denitrifying *Advenella* sp. G7 in the MP digester could also be detrimental to methanogenesis, although their total relative abundance is much lower than the sulfate/sulfite/sulfur-respiring sub-community in the AP reactor (Fig. 6b). For example, our genomic evidence highlights that G6 can utilize organic acids (e.g., acetate, butyrate, benzoate, lactate, pyruvate), alcohols (e.g., ethanol), and some amino acids as carbon and energy sources, while G7 can assimilate acetate, propionate, lactate and pyruvate for growth. Therefore, they can compete with acetoclastic methanogens for acetate (e.g., G23) and with syntrophs for these organic acids and alcohols. The sustainable levels of nitrate (9.4–23.6 mg/L) in the MP bioreactor are probably attributed by the transient introduction of dissolved oxygen during bioreactor feeding.

## Conclusions

The metabolic roles of uncultured microorganisms prevalent in previous phenol-degrading methanogenic bioreactors are predicted by genome-resolved metagenomics. Comparative genomics enriches our view on the microbial syntrophic and competitive interactions in phenol-degrading methanogenic consortia. Revealing a relationship between the formation of distinct but cooperative sulfate/sulfite/sulfur or nitrate/nitrite-reducing sub-communities and deteriorated methanogenic activity justifies biological manipulation to maximize methanogenesis in full-scale anaerobic digesters. While genome-resolved metagenomics shows its power in mining uncultured bacteria, other complementary approaches, such as activity- and cultivation-based ones, are needed to further validate their genome-resolved physico-chemical properties and metabolic pathways.

## Additional file

**Additional file 1: Text S1.** Comparison of *Syntrophorhabdus* genomes. **Figure S1.** The bioinformatics analysis workflow. **Figure S2.** Genome-wide statistics of taxonomic distribution of protein-coding genes in reconstructed genomes. **Figure S3.** Genome comparison between G1 and other sequenced ε-*Proteobacteria*. **Figure S4.** The “Dch-Had-Oah” pathway encoded in *Syntrophorhabdus* genomes constructed from phenol-degrading reactors. **Figure S5.** Key KEGG pathways encoded in the genomes of G3 and G6. **Table S1.** Assembly statistics of the MP and AP metagenomes. **Table S2.** List of 107 essential single-copy marker genes (ESCGs) and 35 conserved clusters of orthologous group markers (COGs). **Table S3.** Genomic information of 23 genomes reconstructed from phenol-degrading metagenomes. **Table S4.** Comparison between uncultured *Sulfurovum*-like G1 and typical sulfur- and/or hydrogen-oxidizing ε-*Proteobacteria*. **Table S5.** Genomic overview and comparison of three draft genomes of *Syntrophorhabdus.* G2 and strain UI both belong to the same species *S. aromaticivorans*, whereas G5 is affiliated with a novel *Syntrophorhabdus* species. NA: not applicable; ND: not detected. **Table S6.** Enzymes encoded by *Cryptanaerobacter* sp. G14 for phenol biodegradation, dissimilatory sulfite reduction, syntrophic propionate oxidation, and pyruvate metabolism. **Table S7.** Key KEGG metabolic pathway enzymes encoded in reconstructed genomes. G1: uncultured ε-*Proteobacterium*; G3: uncultured *Chloroflexi* T78 clade bacterium; G6: *Brachymonas*; G7: *Advenella*; G12: *Syntrophus aciditrophicus*; G15: uncultured *Mycobacterium* species; G21: uncultured *Smithella* species.

## Abbreviations

AAI: average amino acid identity
AdDH: acetaldehyde dehydrogenase
ANI: average nucleotide identity
CRISPRs: clustered regularly interspaced short palindromic repeats
ESCGs: essential single-copy marker genes
HTS: high-throughput sequencing
KEGG: Kyoto Encyclopedia of Genes and Genomes
LCAs: lowest common ancestors
rTCA: reductive tricarboxylic acid cycle
Sox: sulfur-compound oxidation
NRB: nitrate-reducing bacteria
SRB: sulfate-reducing bacteria
IRB: iron-reducing bacteria
DDH: DNA–DNA hybridization
Pps: phenylphosphate synthase
Ppc: phenylphosphate carboxylase
MMC: methylmalonyl-CoA pathway
