# Supplementary material for: Bioreactor microbial ecosystems with differentiated methanogenic phenol biodegradation and competitive metabolic pathways unraveled with genome-resolved metagenomics
Source: Biotechnol Biofuels. 2018 May 11;11:135. doi: 10.1186/s13068-018-1136-6 (PMC5946492; doi:10.1186/s13068-018-1136-6)
Supplement: Supplementary file 1 — Additional file 1: Text S1. Comparison of Syntrophorhabdus genomes. Figure S1. The bioinformatics analysis workflow. Figure S2. Genome-wide statistics of taxonomic distribution of protein-coding genes in reconstructed genomes. Figure S3. Genome comparison between G1 and other sequenced ε-Proteobacteria. Figure S4. The “Dch-Had-Oah” pathway encoded in Syntrophorhabdus genomes constructed from phenol-degrading reactors. Figure S5. Key KEGG pathways encoded in the genomes of G3 and G6. Table S1. Assembly statistics of the MP and AP metagenomes. Table S2. List of 107 essential single-copy marker genes (ESCGs) and 35 conserved clusters of orthologous group markers (COGs). Table S3. Genomic information of 23 genomes reconstructed from phenol-degrading metagenomes. Table S4. Comparison between uncultured Sulfurovum-like G1 and typical sulfur- and/or hydrogen-oxidizing ε-Proteobacteria. Table S5. Genomic overview and comparison of three draft genomes of Syntrophorhabdus. G2 and strain UI both belong to the same species S. aromaticivorans, whereas G5 is affiliated with a novel Syntrophorhabdus species. NA: not applicable; ND: not detected. Table S6. Enzymes encoded by Cryptanaerobacter sp. G14 for phenol biodegradation, dissimilatory sulfite reduction, syntrophic propionate oxidation, and pyruvate metabolism. Table S7. Key KEGG metabolic pathway enzymes encoded in reconstructed genomes. G1: uncultured ε-Proteobacterium; G3: uncultured Chloroflexi T78 clade bacterium; G6: Brachymonas; G7: Advenella; G12: Syntrophus aciditrophicus; G15: uncultured Mycobacterium species; G21: uncultured Smithella species. [file 13068_2018_1136_MOESM1_ESM.docx]

**ADDITIONAL INFORMATION**

**Bioreactor Microbial Ecosystems with Differentiated Methanogenic Phenol Biodegradation and Competitive Metabolic Pathways Unraveled with Genome-resolved Metagenomics**

Feng Ju ^1,2^, Yubo Wang ^1^, Tong Zhang ^1^*

**^1^** Environmental Biotechnology Lab, The University of Hong Kong SAR，China; **^2^** Institute of Advanced Technology, Westlake Institute for Advanced Study, Westlake University, Hangzhou 310064, P. R. China

**This PDF file includes:**

Text s1**:** Comparative genomics of *Syntrophorhabdus* species

**Figure S1** The bioinformatics analysis workflow.

**Figure S2** Genome-wide statistics of taxonomic distribution of protein-coding genes in reconstructed genomes.

**Figure S3** Genome comparison between G1 and other sequenced ε-*Proteobacteria*.

**Figure S4** The “Dch-Had-Oah” pathway encoded in *Syntrophorhabdus* genomes constructed from phenol-degrading reactors.

**Figure S5** Key KEGG pathways encoded in the genomes of G3, G6, and G7. (pdf file, 677 KB)

**Table S1** Assembly statistics of the MP and AP metagenomes.

**Table S2** List of 107 essential single copy marker genes (ESCGs) and 35 conserved clusters of orthologous group markers (COGs).

**Table S3** Genomic information of 23 genomes reconstructed from phenol-degrading metagenomes.

**Table S4** Comparison between uncultured *Sulfurovum*-like G1 and typical sulfur- and/or hydrogen-oxidizing ε-*Proteobacteria*.

**Table S5** Genomic overview and comparison of three draft genomes of *Syntrophorhabdus.* G2 and strain UI both belongto the same species *S. aromaticivorans*, whereas G5 is affiliated with a novel *Syntrophorhabdus* species. NA: not applicable; ND: not detected.

**Table S6** Enzymes encoded by *Cryptanaerobacter* sp. G14 for phenol biodegradation, dissimilatory sulfite reduction, syntrophic propionate oxidation, and pyruvate metabolism.

**Table S7** Key KEGG metabolic pathway enzymes encoded in reconstructed genomes. G1: uncultured ε-*Proteobacterium*; G3: uncultured *Chloroflexi* T78 clade bacterium; G6: *Brachymonas*; G7: *Advenella*; G12: *Syntrophus aciditrophicus*; G15: unclutured *Mycobacterium* species; G21: unclutured *Smithella* species.

### **Text s1: Comparative genomics of *Syntrophorhabdus* species**

G2 and G5 were clustered with multiple environmental 16S clones from previous phenol ([Chen et al 2008](#_ENREF_2)), dimethyl phthalate ([Zhang et al 2009](#_ENREF_38)), and terephthalate ([Wu et al 2001](#_ENREF_37)) degrading anaerobic bioreactor sludge (Fig. 2), suggesting an important role of *Syntrophorhabdus* lineage in aromatic compounds metabolism. The estimated DNA-DNA hybridization value (DDH; 70% threshold), average nucleotide identity (ANI, 95% threshold), average amino acid identity (AAI; 95% threshold) (Table S6), and phylogenetic analysis (Fig. 2) congruously demonstrated that G5 and *S. aromaticivorans* strain UI (DDH: 12.9%; ANI: 77.6%; AAI: 62.7%; 16S similarity: 94.1%) were distinct species, whereas G2 and strain UI (DDH: 79.2%; ANI: 99.8%; AAI: , 98.9%; 16S similarity: 100%) belonged to the same species. A gene-by-gene manual comparison shows that G2, strain UI, and G5 share protein-coding gene cassettes for syntrophic phenol, 4-OHB, and benzoate biodegradation (Fig. 5a, Table S6). This includes (I) phenylphosphate synthase (PpsAB) and phenylphosphate carboxylase (PpcBCCAD) for phenol conversion to 4-OHB, via a phosphorylation-carboxylation pathway (Fig. 5a) utilized by phenol-degrading NRB, SRB, and IRB (Fig. 5b); (II) benzoate-CoA ligase (BCL) for benzoate degradation to benzoyl-CoA; (III) putative 4-OHB CoA ligase (4-BCL) and 4-hydroxybenzoyl CoA reductase (4-HBCR) for 4-OHB degradation to benzoyl-CoA; and (IV) benzoyl-CoA reductase and “Dch-Had-Oah” pathway enzymes for ring cleavage of benzoyl-CoA (Fig. S6a). Moreover, these three *Syntrophorhabdus* genomes have joint characteristic genes indicative of a syntrophic lifestyle, including electron transfer flavoproteins, formate dehydrogenases, as well as an IFO-associated cassette encoding heterodisulfide reductases (HdrABC, electron transfer genes), hydrogenase subunits (HydAEFCDG), and Ion-translocating Fd:NADH oxidoreductase subunits (IfoAB) (Table S6). The IFO-associated cassette may be responsible for a novel reverse electron transport (from NADH to Fd_ox_) for energy conservation in Rnf-lacking syntrophic metabolizers of aromatic compounds, such as strain UI ([Nobu et al 2014](#_ENREF_21)).

### **References**

Chen CL, Wu JH, Liu WT. Identification of important microbial populations in the mesophilic and thermophilic phenol-degrading methanogenic consortia. *Water Res* 2008;**42**: 1963-76.

Nobu MK, Narihiro T, Hideyuki T *et al.* The genome of Syntrophorhabdus aromaticivorans strain UI provides new insights for syntrophic aromatic compound metabolism and electron flow. *Environ Microbiol* 2014.

Wu J-H, Liu W-T, Tseng I-C *et al.* Characterization of microbial consortia in a terephthalate-degrading anaerobic granular sludge system. *Microbiology* 2001;**147**: 373-82.

Zhang T, Liang DW, Fang HHP. Microbial characterization and quantification of an anaerobic sludge degrading dimethyl phthalate. *J Appl Microbiol* 2009;**106**: 296-305.

**Figure S1 The bioinformatics analysis workflow.** The metagenomes with a library insert size of 800 bps are generated in this study, while the amplicoin sequences and metagneomes with a library inset size of 180 bps were generated in our former study (Ju and Zhang et at., 2014).

**Figure S2 Genome-wide statistics of taxonomic distribution of protein-coding genes in reconstructed genomes.** G1: uncultured ε-proteobacterium; G3: *Anaerolineaceae* bacterium of *Chloroflexi* T78 clade; G4: candidate division OP8 bacterium.

**Figure S3 Genome comparison between G1 and other sequenced ε-*Proteobacteria*.** KEGG pathway annotation of 99 publicly-available genomes was downloaded from JGI-IMG database. The principal component analysis was based on abundance matrixes of KOs involved in KEGG pathways for each genome.

**Figure S4 The “Dch-Had-Oah” pathway encoded in *Syntrophorhabdus* genomes constructed from phenol-degrading reactors.** The enzymes that were detectable in all three genomes were filled in green.

**Figure S5** Key KEGG metabolic pathways encoded in the genomes of G3 and G6. A rectangle box filled in light blue indicates that the enzyme is detected in the genome, with corresponding EC number shwon. Green lines show biodegradation pathway. Red, purple and blue open rectangles highlight substrates, intermediates, and end products, respectively. All detected enzyme-coding genes are listed in Data Set S1-G3 and G6. (a) G3: beta-oxidation of butyrate; (b) G6: nitrate/nitrite dentrification; (c) G6: propionate degradation via three different pathways I, II and III.

**Table S1 Assembly statistics of the MP and AP metagenomes.**

|  | **MP** | **AP** |
| --- | --- | --- |
| Enrichment temperature (^o^C) | 37 | 20 |
| Metagenome size (Gbp) | 11.0 | 8.6 |
| Number of reads | 110,083,958 | 85,689,638 |
| Average length of reads (bp) | 100 | 100 |
| Total assembly size (Mbp)^1^ | 172 | 209 |
| N50 (bp)^2^ | 6,519 | 6,612 |
| Max scaffold size (bp) | 423,020 | 980,743 |
| % Reads used in the assembly | 78.9% | 73.3% |
| % Reads mapped to MP scaffolds | 78.9% | 49.73% |

^1^Only scaffolds ≥ 1000 bp were considered.

^2^Calculated by mapping reads to scaffold ≥ 1000 bp using CLC workbench 6.0.

**Table S2 List of 107 essential single copy marker genes (ESCGs) and 35 conserved clusters of orthologous group markers (COGs).**

107 ESCGs

| **Accession ID** | **Name** |
| --- | --- |
| TIGR00344 | Alanine--trna ligase |
| PF00750 | Arginine--trna ligase |
| TIGR00459 | Aspartate--trna ligase |
| TIGR02729 | Obg family gtpase cgta |
| TIGR00152 | Dephospho-coa kinase |
| TIGR00435 | Cysteine--trna ligase |
| TIGR00362 | Chromosomal replication initiator protein dnaa |
| TIGR01391 | DNA primase |
| TIGR02350 | Chaperone protein dnak |
| TIGR00663 | DNA polymerase III, beta subunit |
| TIGR02397 | DNA polymerase III, subunit gamma and tau |
| TIGR03594 | Ribosome-associated gtpase enga |
| TIGR00436 | GTP-binding protein Era |
| TIGR00959 | Signal recognition particle protein |
| TIGR00460 | Methionyl-trna formyltransferase |
| TIGR00496 | Ribosome recycling factor |
| TIGR00064 | Signal recognition particle-docking protein ftsy |
| TIGR00388 | Glycine--trna ligase, alpha subunit |
| TIGR03263 | Guanylate kinase |
| PF01025 | Co-chaperone grpe |
| TIGR01063 | DNA gyrase, A subunit |
| TIGR01059 | DNA gyrase, B subunit |
| TIGR00442 | Histidine--trna ligase |
| TIGR00392 | Isoleucine--trna ligase |
| TIGR00487 | Translation initiation factor IF-2 |
| TIGR00168 | Translation initiation factor IF-3 |
| TIGR01393 | Elongation factor 4 |
| TIGR00396 | Leucine--trna ligase |
| TIGR00575 | DNA ligase, NAD-dependent |
| TIGR00420 | Trna(5-methylaminomethyl-2-thiouridylate)-methyltransferase |
| PF01795 | Mraw methylase family |
| TIGR01953 | Transcription termination factor nusa |
| TIGR00922 | Transcription termination/antitermination factor nusg |
| PF00162 | Phosphoglycerate kinase |
| TIGR00468 | Phenylalanine--trna ligase, alpha subunit |
| TIGR00472 | Phenylalanine--trna ligase, beta subunit |
| TIGR00019 | Peptide chain release factor 1 |
| TIGR00408 | Proline--trna ligase |
| TIGR00409 | Proline--trna ligase |
| TIGR00337 | CTP synthase |
| TIGR02012 | Protein reca |
| TIGR00082 | Ribosome-binding factor A |
| TIGR02191 | Ribonuclease III |
| TIGR01169 | Ribosomal protein L1 |
| TIGR01171 | Ribosomal protein L2 |
| PF00297 | Ribosomal protein L3 |
| PF00573 | Ribosomal protein L4/L1 family |
| PF00281 | Ribosomal protein L5 |
| PF00347 | Ribosomal protein L6 |
| TIGR00158 | Ribosomal protein L9 |
| PF00466 | Ribosomal protein L10 |
| TIGR01632 | Ribosomal protein L11 |
| TIGR00855 | Ribosomal protein L7/L12 |
| TIGR01066 | Ribosomal protein L13 |
| TIGR01067 | Ribosomal protein L14 |
| TIGR01071 | Ribosomal protein L15 |
| TIGR01164 | Ribosomal protein L16 |
| TIGR00059 | Ribosomal protein L17 |
| TIGR00060 | Ribosomal protein L18 |
| TIGR01024 | Ribosomal protein L19 |
| TIGR01032 | Ribosomal protein L20 |
| TIGR00061 | Ribosomal protein L21 |
| TIGR01044 | Ribosomal protein L22 |
| PF00276 | Ribosomal protein L23 |
| TIGR01079 | Ribosomal protein L24 |
| TIGR00062 | Ribosomal protein L27 |
| TIGR00009 | Ribosomal protein L28 |
| TIGR00012 | Ribosomal protein L29 |
| TIGR01031 | Ribosomal protein L32 |
| TIGR01030 | Ribosomal protein L34 |
| TIGR00001 | Ribosomal protein L35 |
| TIGR02027 | DNA-directed RNA polymerase, alpha subunit |
| TIGR02013 | DNA-directed RNA polymerase, beta subunit |
| TIGR02386 | DNA-directed RNA polymerase, beta' subunit |
| TIGR01011 | Ribosomal protein S2 |
| TIGR01009 | Ribosomal protein S3 |
| TIGR01017 | Ribosomal protein S4 |
| TIGR01021 | Ribosomal protein S5 |
| TIGR00166 | Ribosomal protein S6 |
| TIGR01029 | Ribosomal protein S7 |
| PF00410 | Ribosomal protein S8 |
| PF00380 | Ribosomal protein S9 |
| TIGR01049 | Ribosomal protein S10 |
| PF00411 | Ribosomal protein S11 |
| TIGR00981 | Ribosomal protein S12 |
| PF00416 | Ribosomal protein s13p/s18e |
| TIGR00952 | Ribosomal protein S15 |
| TIGR00002 | Ribosomal protein S16 |
| PF00366 | Ribosomal protein S17 |
| TIGR00165 | Ribosomal protein S18 |
| TIGR01050 | Ribosomal protein S19 |
| TIGR00029 | Ribosomal protein S20 |
| TIGR00963 | Preprotein translocase, seca subunit |
| TIGR00964 | Preprotein translocase, sece subunit |
| TIGR00810 | Preprotein translocase, secg subunit |
| TIGR00967 | Preprotein translocase, secy subunit |
| TIGR00414 | Serine--trna ligase |
| TIGR00086 | Ssra-binding protein |
| TIGR00418 | Threonine--trna ligase |
| TIGR00115 | Trigger factor |
| TIGR02432 | Trna(Ile)-lysidine synthetase |
| TIGR00116 | Translation elongation factor Ts |
| TIGR00234 | Tyrosine--trna ligase |
| TIGR00631 | Excinuclease ABC subunit B |
| TIGR00422 | Valine--trna ligase |
| TIGR00043 | Probable rrna maturation factor ybey |
| TIGR00092 | GTP-binding protein ychf |

35 COGs

| OG markers | Name |
| --- | --- |
| COG0012 | Predicted GTPase, probable translation factor |
| COG0016 | Phenylalanyl-tRNA synthetase alpha subunit |
| COG0048 | Ribosomal protein S12 |
| COG0049 | Ribosomal protein S7 |
| COG0052 | Ribosomal protein S2 |
| COG0080 | Ribosomal protein L11 |
| COG0081 | Ribosomal protein L1 |
| COG0085 | DNA-directed RNA polymerase, beta subunit/140 kD subunit |
| COG0087 | Ribosomal protein L3 |
| COG0088 | Ribosomal protein L4 |
| COG0090 | Ribosomal protein L2 |
| COG0091 | Ribosomal protein L22 |
| COG0092 | Ribosomal protein S3 |
| COG0093 | Ribosomal protein L14 |
| COG0094 | Ribosomal protein L5 |
| COG0096 | Ribosomal protein S8 |
| COG0097 | Ribosomal protein L6P/L9E |
| COG0098 | Ribosomal protein S5 |
| COG0099 | Ribosomal protein S13 |
| COG0100 | Ribosomal protein S11 |
| COG0102 | Ribosomal protein L13 |
| COG0103 | Ribosomal protein S9 |
| COG0124 | Histidyl-tRNA synthetase |
| COG0184 | Ribosomal protein S15P/S13E |
| COG0185 | Ribosomal protein S19 |
| COG0186 | Ribosomal protein S17 |
| COG0197 | Ribosomal protein L16/L10E |
| COG0200 | Ribosomal protein L15 |
| COG0201 | Preprotein translocase subunit SecY |
| COG0256 | Ribosomal protein L18 |
| COG0495 | Leucyl-tRNA synthetase |
| COG0522 | Ribosomal protein S4 and related proteins |
| COG0525 | Valyl-tRNA synthetase |
| COG0533 | Metal-dependent proteases with possible chaperone activity |
| COG0541 | Signal recognition particle GTPase |

**Table S3 Genomic information of 23 genomes reconstructed from phenol-degrading metagenomes.** The completeness and potential contamination of draft genomes are estimated by 107 essential single copy marker genes (ESCGs) and 35 conserved clusters of orthologous groups markers, as listed in Table S3. Gs: genome size (MB); Ns: number of scaffolds; Ls: average length of scaffolds (bp); cov.: genome coverage in metagenome; C(%): genome completeness; R(%): genome contamination.

| **ID** | **Genome name** | **IMG genome ID** | **NBCI taxon ID** | **Taxonomy affiliation**  **(Phylum; Lowest rank assigned)** | **Gs** | **Ns** | **Ls** | **GC (%)** | **MP-cov.**  **(37^o^C)** | **AP-cov.**  **(20^o^C)** | **Total**  **ESCGs** | **Uni.**  **ESCGs** | **C(%)** | **R(%)** |
| --- | --- | --- | --- | --- | --- | --- | --- | --- | --- | --- | --- | --- | --- | --- |
| 1 | *Sulfurovum-like* G1 | 2597489806 | 1578167 | *Proteobacteria; Epsilonproteobacteria* | 1.78 | 27 | 65754 | 37.0 | 74.6 | 295.5 | 107 | 106 | 99.1 | 0.9 |
| 2 | *Syntrophorhabdus aromaticivorans* G2 | 2599185327 | 328301 | *Proteobacteria; S. Aromaticivorans* | 2.90 | 133 | 21836 | 51.9 | 634.5 | 427.1 | 101 | 99 | 92.5 | 1.9 |
| 3 | *Anaerolineaceae bacterium* G3 | 2599185328 | 1580604 | *Chloroflexi;* T78 *clade* | 1.84 | 44 | 41798 | 46.9 | 516.3 | 14.4 | 101 | 99 | 92.5 | 1.9 |
| 4 | *Aminicenantes* bacterium G4 | 2599185329 | 1578176 | *Aminicenantes; Uncultured* OP8 | 2.30 | 55 | 41874 | 45.6 | 51.7 | 6.0 | 98 | 96 | 89.7 | 1.9 |
| 5 | *Syntrophorhabdus sp.* G5 | 2600254908 | 1578168 | *Proteobacteria; Syntrophorhabdus* | 3.17 | 77 | 41142 | 44.0 | 45.1 | 2.6 | 95 | 94 | 87.9 | 0.9 |
| 6 | *Brachymonas denitrificans* G6 | 2600254909 | 28220 | *Proteobacteria; B. Denitrificans* | 2.57 | 44 | 58343 | 64.6 | 95.6 | 0.5 | 94 | 90 | 84.1 | 3.7 |
| 7 | *Advenella faeciporci* G7 | 2600254910 | 797535 | *Proteobacteria; A. Faeciporci* | 2.91 | 28 | 104013 | 49.8 | 46.7 | 0.8 | 106 | 102 | 95.3 | 3.7 |
| 8 | *Methanobacterium sp.* G8 | 2602042054 | 1578169 | *Euryarchaeota; Methanobacterium* | 1.72 | 35 | 49174 | 37.9 | 38.1 | 22.5 | 31 | 31 | 88.6 | 0.0 |
| 9 | *Synergistaceae* bacterium G9 | 2602042055 | 1578178 | *Synergistetes; Synergistaceae (100%)* | 1.90 | 17 | 111970 | 56.1 | 25.4 | 0.1 | 101 | 97 | 90.7 | 3.7 |
| 10 | *Methanolinea sp.* G10 | 2602042056 | 1578170 | *Euryarchaeota; Methanolinea* | 2.21 | 56 | 39540 | 57.3 | 24.3 | 0.3 | 31 | 31 | 88.6 | 0.0 |
| 11 | *Sphingobacteriales* bacterium G11 | 2602042057 | 1578179 | *Bacteroidetes; Sphingobacteriales* | 2.40 | 84 | 28516 | 39.7 | 22.1 | 4.5 | 99 | 98 | 91.6 | 0.9 |
| 12 | *Syntrophus aciditrophicus* G12 | 2600254911 | 316277 | *Proteobacteria; S. Aciditrophicus* | 2.43 | 65 | 37393 | 51.8 | 48.1 | 9.4 | 97 | 95 | 88.8 | 1.9 |
| 13 | *Synergistaceae bacterium* G13 | 2602042058 | 1578177 | *Synergistetes; Synergistaceae* | 2.48 | 93 | 26705 | 60.0 | 13.8 | 9.9 | 94 | 92 | 86.0 | 1.9 |
| 14 | *Cryptanaerobacter phenolicus* G14 | 2600254912 | 1578171 | *Proteobacteria; C. Phenolicus* | 2.97 | 127 | 23401 | 48.2 | 13.6 | 68.3 | 91 | 91 | 85.0 | 0.0 |
| 15 | *Mycobacterium sp.* G15 | 2600254913 | 1578172 | *Actinobacteria; Mycobacterium* | 4.91 | 159 | 30894 | 68.6 | 13.7 | 27.1 | 96 | 96 | 89.7 | 0.0 |
| 16 | *Anaerolinaceae* bacterium G16 | 2602042059 | 1578173 | *Chloroflexi; Anaerolinaceae (92%)* | 1.88 | 103 | 18279 | 52.2 | 21.8 | 12.7 | 91 | 90 | 84.1 | 0.9 |
| 17 | *Anaerolineaceae* bacterium G17 | 2602042060 | 1578181 | *Chloroflexi; Anaerolinaceae (85%)* | 2.16 | 130 | 16641 | 52.5 | 13.5 | 0.9 | 94 | 93 | 86.9 | 0.9 |
| 18 | *Anaerolineaceae* bacterium G18 | 2602042061 | 1578182 | *Chloroflexi; Anaerolinaceae (86%)* | 2.13 | 38 | 56121 | 50.8 | 10.8 | 20.0 | 101 | 99 | 92.5 | 1.9 |
| 19 | *Bacteroidales* bacterium G19 | 2602042062 | 1578180 | *Bacteroidetes; Bacteroidales* | 2.70 | 75 | 36050 | 49.6 | 10.8 | 16.5 | 97 | 96 | 89.7 | 0.9 |
| 20 | *Syntrophus sp.* G20 | 2600254914 | 1578174 | *Proteobacteria; Syntrophus* | 2.35 | 132 | 17784 | 55.7 | 10.3 | 58.6 | 81 | 79 | 73.8 | 1.9 |
| 21 | *Smithella sp.* G21 | 2602042063 | 1578175 | *Proteobacteria; Smithella* | 2.42 | 106 | 22845 | 55.3 | 5.8 | 20.6 | 100 | 99 | 92.5 | 0.9 |
| 22 | *Desulfovibrio aminophilus* G22 | 2602042064 | 81425 | *Proteobacteria; D. Aminophilus* | 2.64 | 240 | 11012 | 66.8 | 5.8 | 11.2 | 92 | 92 | 86.0 | 0.0 |
| 23 | *Methanosaeta concilii* G23 | 2600254915 | 2223 | *Euryarchaeota; M. Concilii* | 2.08 | 220 | 9432 | 52.8 | 401.5 | 187.1 | 21 | 20 | 60.0 | 2.9 |

**Table S4 Comparison between uncultured ε-Proteobacterium G1 and typical sulfur- and/or hydrogen-oxidizing ε-*Proteobacteria*.** Abbreviations: Uncl., Uncultured; Ref., References; Y, Yes; N, No; G, Genes found.

| **ID** | **Source** | **Order, Family** | **Organism** | **Autotroph** | **Aerobic** | **Anaerobic** | **Sox multienzyme system** | **H_2_ oxidation** | **NO_3_ reduction** | **N_2_ fixation** | **Genome** | **Genome**  **size (Mb)** | **GC**  **(%)** |
| --- | --- | --- | --- | --- | --- | --- | --- | --- | --- | --- | --- | --- | --- |
| **1** | **Anaerobic reactors**  **( This study)** | **Uncl. ε-Proteobacteria** | ***Sulfurovum*-like G1** | **G** | **G** | **Y** | **N** | **G** | **G** | **G** | **Draft (uncl.)** | 1.78 | 37.1 |
| 2 | Microbial fuel cell | *Helicobacteraceae* | *Arcobacter* sp. L | - (+  heterotroph) | Y | - | Y | - | - | **Y** | Y | 2.95 | 26.6 |
| **3** | Anaerobic enrichment | *Helicobacteraceae* | ***Sulfurospirillum deleyianum*** | Mixotroph | Y | Y | **N** | Y | Y | **N** | Y | 2.31 | 39.0 |
| 4 | Rifle aquifer sediment | *Helicobacteraceae* | Ca. *Sulfuricurvum* RIFRC-1 | G | G | Y | soxXYZAB | G | G | **G** | Y (uncl.) | 2.36 | 43.4 |
| 5 |  | *Helicobacteraceae* | Ca. *Sulfurimonas* sp. r9c3 | G | G | Y | soxCDYZ | - | G | N | Draft (uncl.) | - | 36.9 |
| 6 |  | Uncl. *ε-*Proteobacteria | Ca. *Sulfurovum* sp. r9c2 | G | G | Y | soxCDYZ | - | G | N | Draft (uncl.) | - | 40.1 |
| 7 | Deep sea hydrothermal vent | *Helicobacteraceae* | *Sulfurimonas autotrophica* | Y | Y | N | soxCDXAYZ | N | N | N | Y | 2.15 | 35.2 |
| 8 |  | Uncl. *ε-*Proteobacteria | *Sulfurovum* sp. NBC37-1 | Y | Y | Y | soxXYZABCDYZHFF | Y | Y | N | Y | 2.56 | 43.8 |
| 9 |  | Uncl. *ε-*Proteobacteria | *Sulfurovum lithotrophicum* | Y | Y | Y | Y | - | Y | N | N | - | 48.0 |
| 10 |  | Uncl. *ε-*Proteobacteria | *Nitratiruptor* sp. SB155-2 | Y | Y | Y | soxXYZABFLYZH | Y | Y | N | Y | 1.88 | 39.7 |
| **11** |  | *Nautiliaceae* | ***Nautilia profundicola* sp. nov.** | Y | Y | Y | **N** | Y | Y | **Y** | Y | 1.68 | 34.0 |
| 12 |  | Uncl. *ε-*Proteobacteria | ***Nitratifractor salsuginis*** DSM 16511 | Y | Y | Y | **soxXYZAB** | Y | Y | **Y** | Y | 2.10 | 54.0 |
| 13 | Coastal sediment | *Helicobacteraceae* | *Sulfurimonas hongkongensis* AST-10 | Y | N | Y | Y | Y | Y | N | Draft | 2.30 | 34.9 |
| 14 | Marine sediment | *Helicobacteraceae* | *Sulfurimonas denitrificans* | Y | G | Y | soxXYZABCDYZH | G | Y | N | Y | 2.20 | 34.5 |
| 15 |  | *Helicobacteraceae* | *Sulfurimonas gotlandica* GD1 | Y (+  heterotroph) | G | Y | soxCDXAYZ | Y | Y | N | Y | 2.95 | 33.6 |
| 16 |  | *Helicobacteraceae* | ***Thiovulum sp.* ES** | Y | Y | N | **N** | - | G | **N** | Draft (uncl.) | 2.08 | 33.0 |
| 17 |  | Uncl. *ε-*Proteobacteria | *Sulfurovum* sp. AR | G | - | - | soxYZABCD | - | G | N | Draft | 2.12 | 39.4 |
| 18 | Underground crude-oil storage | *Helicobacteraceae* | *Sulfuricurvum kujiense* | Y | Y | Y | **soxXYZAB** | Y | Y | **G** | Y | 2.57 | 44.6 |
| 19 | Aqueous, environments, | *Helicobacteraceae* | Arcobacter butzleri RM4018 | Y (+  heterotroph) | Y | Y | soxXYZABCD | Y | Y | N | Y | 2.34 | 27.0 |

References: (1) This study; (2) ([Fedorovich et al 2009](#_ENREF_4), [Toh et al 2011](#_ENREF_33)); (3) ([Eisenmann et al 1995](#_ENREF_3), [Sikorski et al 2010a](#_ENREF_28)); (4) ([Handley et al 2014](#_ENREF_9)); (5) ([Handley et al 2013](#_ENREF_8)); (6) ([Handley et al 2013](#_ENREF_8)); (7) ([Sikorski et al 2010b](#_ENREF_29)); (8) ([Nakagawa et al 2007](#_ENREF_20)); (9) ([Inagaki et al 2004](#_ENREF_11)); (10) ([Nakagawa et al 2007](#_ENREF_20)); (11) ([Smith et al 2008](#_ENREF_30)) ; (12) ([Nakagawa et al 2005](#_ENREF_19)); (13) ([Cai et al 2014](#_ENREF_1)); (14) ([Sievert et al 2008](#_ENREF_27)); (15) ([Grote et al 2012](#_ENREF_6), [Labrenz et al 2013](#_ENREF_14)); (16) ([Wirsen and Jannasch 1978](#_ENREF_36)); (17) ([Park et al 2012](#_ENREF_23)); (18) ([Han et al 2012](#_ENREF_7), [Kodama and Watanabe 2004](#_ENREF_13)) ; (19) ([Miller et al 2007](#_ENREF_18))

**Table S5** Genomic overview and comparison of three draft genomes of *Syntrophorhabdus.* G2 and strain UI both belongto the same species *S. aromaticivorans*, whereas G5 is affiliated with a novel *Syntrophorhabdus* species. NA: not applicable; ND: not detected.

|  | **Description** | **Genome overview** | | | **Genome comparison** | |
| --- | --- | --- | --- | --- | --- | --- |
|  |  | **G2** | **G5** | **Strain UI** | **G2 vs. UI** | **G5 vs. UI** |
|  | IMG taxon ID | 2599185327 | 2600254908 | 2509601044 | NA | NA |
| **Genome statistics** | Genome sizes (bases) | 2904229 | 3169307 | 3759482 | NA | NA |
|  | No. of protein-coding genes | 2899 | 3178 | 3632 | NA | NA |
|  | GC content | 51.02% | 43.38% | 51.93% | NA | NA |
|  | ESCGs-based completeness estimate | 92.5% | 87.9% | 99.1% | NA | NA |
|  | DNA-DNA hybridization (DDH) estimate (%)^1^ | NA | NA | NA | 79.2% | 12.9% |
|  | Average nucleotide identity, ANI (number of fragments) ^2^ | NA | NA | NA | 99.8% (12298) | 77.6% (219) |
|  | Average amino acid identity, AAI (number of ORFs) ^3^ | NA | NA | NA | 98.9% (2810) | 62.7%(2440) |
| **Genes for biodegradation of aromatic compounds** | Phenylphosphate synthase subunits (PpsAB) | 2600081325-24 | 2600282236-35 | 2509869326-25 | 100% | 72.3-74.9% |
|  | Phenylphosphate carboxylase subunits (PpcBCCAD) | 2600081318-14 | 2600282229-25 | 2509869319-15 | 99.8-100% | 65.5-82.2% |
|  | Putative UbiX-like carboxylase (PpcY) | 2600081313 | 2600282223 | 2509869314 | 100% | 81.5% |
|  | Putative UbiD-like carboxylase (PpcX) | 2600081312 | 2600282224 | 2509869313 | 99.5% | 65.3% |
|  | Benzoate-CoA ligase (BCL) | 2600083782 | 2600281121 | 2509867425 | 100% | 46.0% |
|  | 4-hydroxybenzoate CoA ligase (4-BCL) | 2600083782  2600082555 | 2600281121  2600282172 | 2509867425  2509869147 | 100% | 43.5-73.1% |
|  | Putative 4-hydroxybenzoyl CoA reductase (4-HBCR) | 2600081536-34  2600082688-86 | 2600280987-89  2600281655-57 | 2509867503-01  2509868729-27 | 95.9-100% | 34.9-80.1 |
|  | Non-ATP-dependent Benzoyl-CoA reductase (BCR) | 2600083970-71 2600081569-65  2600081973-72 | 2600281835-36  2600282020-16  2600283435-36 | 2509867337-36  2509867335-32  2509869887-88 | 100% | 43.6-90.5% |
|  | Dch-Had-Oah pathway enzymes | 2600083892-90 | 2600280422-20 | 2509867635-34 | 100% | 85.5-91.0% |
|  | Electron transfer flavoproteins subunits | 2600083403-04 | 2600283196-95 | 2509869860-61 | 99.6-99.7% | 85.3-89.3% |
|  | Formate dehydrogenase or its activators | 2600083669  2600082990  2600081570-71 | 2600280660  2600282021 | 2509867405  2509867642  2509867331-30 | 99.3-100% | 33.3-76.5% |
|  | Hydrogenase maturation protein (Hyd A/E/F/C/D/G) | 2600083824-30  2600084083-84  2600083960 | 2600282856-55  2600282858 | 2509868910-16  2509868919-18  2509867814 | 97.2-100% | 75.5-75.7% |
|  | Heterodisulfide reductases subunits (HdrABC) | 2600083666-68  2600083453-51 | 2600280659-57  2600282240-38 | 2509867402-04  2509867112-14 | 100% | 65.4-92.3% |
|  | Ion-translocating Fd:NADH oxidoreductase (IfoAB) | 2600083456-57 | 2600282243-44 | 2509867109-08 | 99.4-100% | 75.9-89.6% |
| **Beta-Lactam and Penicillin resistance** | Arabinose efflux permease | 2600081489 | 2600281927 | 2509869083 | 100% | 73.6% |
|  | Penicillin-binding proteins ( EC:2.4.1.-,EC:3.4.- ) | 2600082337  2600083204  2600081814 | 2600282337  2600282911-12  2600283325-27 | 2509866634  2509867011  2509868961 | 100% | 43.7-82.6 |
|  | Penicillin-binding protein 2 | 2600081814 | 2600283327 | 2509868961 | 100% | 78.6% |
|  | Beta-lactamase and other penicillin binding proteins | 2600081351 | ND | 2509869352 | 100% | NA |
|  | Outer membrane protein | 2600081787 | 2600282525 | 2509869832 | 99.6% | 65.5% |
|  | RND family efflux transporter, MFP subunit | 2600081441  2600083627 | 2600280704  2600281667 | 2509868407  2509869802 | 87.2-99.8% | 27.9-70.4 |
|  | The Hydrophobe/Amphiphile Efflux-1 (HAE1) Family | 2600081440 | 2600280705 | 2509868408 | 95.3% | 85.6% |
|  | Efflux transporter, outer membrane factor lipoprotein | 2600081439  2600083629 | 2600280706  2600282525 | 2509868409  2509869800 | 99.4-100% | 23.1-73.1% |
|  | ABC-type dipeptide/oligopeptide transport systems | 2600083802-03 | 2600281091-92 | 2509866980-79 | 99.7% | 69.9-79.3% |

^1^DDH was caculated by GGDC 2.0 web server at <http://ggdc.dsmz.de/distcalc2.php> using Formula 1; ^2^ANI was calculated based on reciprocal best hits from online ANI calculator with default settings (<http://enve-omics.ce.gatech.edu/ani/>); ^3^AAI was calculated based on reciprocal best hits from local BLASTP search at a maximum e-value of 1e-5.

**Table S6 Enzymes encoded by *Cryptanaerobacter* sp. G14 for phenol biodegradation, dissimilatory sulfite reduction, syntrophic propionate oxidation, and pyruvate metabolism.** PTH: *Pelotomaculum thermopropionicum* SI.

| **ID** | **Functional categories** | **IMG gene ID** | **Functional annotation of protein-coding genes** | **Organism** | **ID, bitscore** |
| --- | --- | --- | --- | --- | --- |
| **Phenol degradation to Benzoate** | Phenol (de-)carboxylation to 4-hydroxybenzoate | 2600292530 | 4-hydroxybenzoate decarboxylase, subunit B | Sedimentibacter hydroxybenzoicus | 51%, 196 |
|  |  | 2600292531 | 4-hydroxybenzoate decarboxylase, subunit C | Sedimentibacter hydroxybenzoicus | 58%, 547 |
|  |  | 2600292532 | Putative 4-hydroxybenzoate decarboxylase, subunit D | Sedimentibacter hydroxybenzoicus | 40%, 51 |
|  | 4-hydroxybenzoate (OHB) to Benzoate (via Benzoyl-CoA) | 2600292885 | Putative 4-hydroxybenzoate-CoA ligases (4-BCL) | Pelotomaculum thermopropionicum | 79%, 1170 |
|  |  | 2600294143 | Putative 4-hydroxybenzoyl-CoA reductase (4-HBCR) | Desulfotomaculum gibsoniae | 59%, 331 |
|  |  | 2600294144 | Putative 4-hydroxybenzoyl-CoA reductase (4-HBCR) | Desulfotomaculum gibsoniae | 68%, 232 |
|  |  | 2600294302 | Benzoate-CoA ligase (EC 6.2.1.25) (BCL) | Desulfotomaculum gibsoniae | 65%, 714 |
|  | Benzoyl-CoA to Cyclohex- 1,5-diene-1-carboxyl-CoA | 2600294447 | Benzoyl-CoA reductase, bcrc/badd/hgdb | Desulfotomaculum acetoxidans | 67%, 529 |
|  |  | 2600294518 | Benzoyl-CoA reductase, bcrc/badd/hgdb | Pelotomaculum thermopropionicum | 77%, 684 |
|  |  |  |  |  |  |
| **MMC pathway** | Propionate to Propionate-CoA | 2600292885 | Acetyl-coenzyme A synthetase (EC 6.2.1.1) | PTH, PTH_2131 | 79%, 1117 |
|  |  | 2600292803 | Propionate CoA-transferase (EC 2.8.3.1) | PTH, PTH_1541 | 66%, 694 |
|  | Propionate-CoA to (R)-Methyl-malonyl-CoA (MMC) | [2600292333](https://img.jgi.doe.gov/cgi-bin/er/main.cgi?section=GeneDetail&page=geneDetail&gene_oid=2600292333) | Acetyl-CoA carboxylase, carboxyltransferase component | PTH, PTH_1364 | 83%, 890 |
|  |  | [2600292332](https://img.jgi.doe.gov/cgi-bin/er/main.cgi?section=GeneDetail&page=geneDetail&gene_oid=2600292332) | Methylmalonyl-CoA epimerase (EC 5.1.99.1) | PTH, PTH_1363 | 89%, 231 |
|  | (R)-Methyl-malonyl-CoA to Succinate | 2600292330 | Methylmalonyl-CoA mutase N-terminal domain | PTH, PTH_1361 | 83%, 890 |
|  |  | 2600292331 | Methylmalonyl-CoA mutase C-terminal domain | PTH, PTH_1362 | 87%, 231 |
|  |  | 2600292328 | Succinyl-CoA synthetase, beta subunit | PTH, PTH_1358 | 78%, 594 |
|  |  | 2600292329 | Succinyl-CoA synthetase, alpha subunit | PTH, PTH_1359 | 80%, 259 |
|  |  |  |  |  |  |
| **Energy conservation system** | Heterodisulfide reductase (Hdr)-associated putative iontranslocating  ferredoxin:NADH oxidoreductase (Hdr-Ifo) | 2600291645 | Putative heterodisulfide reductase, subunit C | PTH, PTH_1413 | 76%, 320 |
|  |  | 2600291646 | Heterodisulfide reductase, subunit B | PTH, PTH_1412 | 74%, 450 |
|  |  | 2600291647 | Putative heterodisulfide reductase | PTH, PTH_1411 | 85%, 356 |
|  |  | 2600291648 | Heterodisulfide reductase, subunit A | PTH, PTH_1410 | 87%, 843 |
|  |  | 2600291649 | Methyl-viologen-reducing hydrogenase | PTH, PTH_1409 | 85%, 129 |
|  |  | 2600291650 | Methyl-viologen-reducing hydrogenase | PTH, PTH_1408 | 74%, 147 |
|  |  | 2600291651 | Puative hydrogenase | PTH, PTH_1407 | 88%, 98 |
|  |  | 2600291652 | Putative ion-translocating Fd:NADH oxidoreductase | PTH, PTH_1406 | 79%, 358 |
|  |  | 2600291653 | Putative ion-translocating Fd:NADH oxidoreductase | PTH, PTH_1405 | 72%, 470 |
|  |  |  |  |  |  |
| **Pyruvate metabolism** | Succinate to Fumarate | 2600294405 | Succinate dehydrogenase subunit C (EC 1.3.5.1) | PTH, PTH_1016 | 74%, 355 |
|  |  | 2600294406 | Succinate dehydrogenase or fumarate reductase | PTH, PTH_1017 | 88%, 1121 |
|  |  | 2600294407 | Succinate dehydrogenase or fumarate reductase | PTH, PTH_1018 | 85%, 456 |
|  | Fumarate to Oxaloacetate (via malate) | 2600292335 | Methylmalonyl-coa decarboxylase | PTH, PTH_1366 | 81%, 119 |
|  |  | 2600292336 | Malate dehydrogenase (NAD) (EC 1.1.1.37) | PTH, PTH_1367 | 83%, 524 |
|  |  | 2600292336 | Malate dehydrogenase (NAD) (EC 1.1.1.37) | PTH, PTH_1367 | 83%, 524 |
|  | Propionyl-CoA to Oxaloacetate | 2600292333 | Methylmalonyl-coa decarboxylase, alpha subunit | PTH, PTH_1364 | 83%, 890 |
|  |  | 2600292334 | Methylmalonyl-coa decarboxylase, epsilon subunit | PTH, PTH_1365 | 66%, 80 |
|  |  | 2600292335 | Methylmalonyl-coa decarboxylase, gamma subunit | PTH, PTH_1366 | 81%, 119 |
|  |  | 2600292337 | Pyruvate/oxaloacetate carboxyltransferase | PTH, PTH_1368 | 88%, 879 |
|  | Oxaloacetate to Pyruvate | 2600291959 | Acetyl-coa carboxylase, biotin carboxyl carrier protein | PTH, PTH_1174 | 69%, 923 |
|  |  | 2600291960 | Acetyl-coa carboxylase, biotin carboxylase subunit | PTH, PTH_1175 | 79%, 758 |
|  | Lactate to Pyruvate | 2600292474 | L-lactate dehydrogenase (EC 1.1.1.27) | PTH, PTH_0747 | 80%, 508 |
|  | Pyruvate to Acetate (via Acetyl-CoA) | 2600293798 | Pyruvate-formate lyase-activating enzyme | Pelotomaculum thermopropionicum | 69%, 598 |
|  |  | 2600292338 | Pyruvate:ferredoxin oxidoreductase | PTH, PTH_1369 | 72%, 1750 |
|  |  | 2600291793 | Acyl-coa synthetases (EC 6.2.1.1) | Pelotomaculum thermopropionicum | 71%, 661 |
|  |  | 2600292807 | Acyl-coa synthetases (EC 6.2.1.1) | Heliobacterium modesticaldum | 60%, 615 |
|  |  | 2600294563 | Acyl-coa synthetases (EC 6.2.1.1) | Thaumarchaeota archaeon | 43%, 331 |
|  |  | 2600294576 | Acyl-coa synthetases (EC 6.2.1.1) | Nodularia spumigena | 44%, 322 |
| **Propionate to Acetate** | Propionate to Acetate (by propionate CoA-transferase) | 2600292865 | Acyl coa:acetate/3-ketoacid coa transferase, beta subunit | PTH, PTH_2042 | 93%, 478 |
|  |  | 2600292866 | Acyl coa:acetate/3-ketoacid coa transferase, alpha subunit | PTH, PTH_2043 | 87%, 663 |
|  |  | 2600292867 | Acyl coa:acetate/3-ketoacid coa transferase, alpha subunit | PTH, PTH_2044 | 83%, 604 |
|  |  | 2600292868 | Acyl coa:acetate/3-ketoacid coa transferase, beta subunit | PTH, PTH_2045 | 92%, 508 |
| **Alcohol utilization** | Ethanol to Acetyl-CoA | 2600294099 | Bifunctional acetaldehyde dehydrogenase /alcohol dehydrogenase | PTH, PTH_1584 | 78%,1409 |
|  | Alcohol dehydrogenation | 2600293222 | Alcohol dehydrogenase IV | PTH, PTH_0606 | 70%, 575 |
|  |  | 2600293227 | Alcohol dehydrogenase IV | PTH, PTH_0606 | 84%, 511 |
|  |  | 2600293412 | Alcohol dehydrogenases, class III | Clostridium sp. | 62%, 461 |
|  |  | 2600294107 | Alcohol dehydrogenase groes-like domain | Pelotomaculum thermopropionicum | 72%, 87 |
|  |  | 2600294099 | Bifunctional acetaldehyde-coa/alcohol dehydrogenase | PTH, PTH_1584 | 78%,1409 |
|  |  | 2600294504 | Aldehyde dehydrogenase, molybdopterin-binding domain | Clostridium tetani | 56%, 325 |
|  |  |  |  |  |  |
| **Hydrogen generation & utilization** | Menaquinone ↔ H_2_  (Ni-Fe hydrogenases) | 2600293465 | Hypa/hybf (regulating hydrogenase expression) | Desulfotomaculum gibsonia | 35%, 69 |
|  |  | 2600293466 | Hydrogenase accessory protein hypb | Desulfotomaculum carboxydivorans | 56%, 266 |
|  |  | 2600293467 | [nife] hydrogenase maturation protein hypf | Desulfotomaculum carboxydivorans | 45%, 662 |
|  |  | 2600293468 | Hydrogenase assembly chaperone hypc/hupf | Desulfitobacterium sp. | 48%, 55 |
|  |  | 2600293469 | Hydrogenase expression/formation protein hypd | Desulfotomaculum carboxydivorans | 61%, 262 |
|  |  | 2600293470 | Hydrogenase expression/formation protein hype | Sulfurihydrogenibium sp. | 51%, 313 |
|  | NADH ↔H_2_  (Iron-only hydrogenases) | 2600291795 | NAD(P)-dependent iron-only hydrogenase catalytic subunit | PTH_1377 | 87%, 1120 |
|  |  | 2600291796 | NAD(P)-dependent iron-only hydrogenase diaphorase component flavoprotein | PTH, PTH_1378 | 80%, 1046 |
|  |  | 2600291797 | NADH:ubiquinone oxidoreductase 24 kd subunit | PTH, PTH_1379 | 60%, 226 |
|  | Ferredoxin ↔ H_2_  (Iron-only hydrogenases) | 2600291614 | Putative iron-only hydrogenase system regulator | PTH, PTH_0984 | 76%, 743 |
|  |  | 2600291613 | Iron-only hydrogenase maturation protein hydf | PTH, PTH_0983 | 66%, 459 |
|  |  | 2600291612 | Iron-only hydrogenase maturation protein hyde | PTH, PTH_0982 | 81%, 686 |
|  |  | 2600291611 | Iron-only hydrogenase maturation protein hydg | PTH, PTH_0980 | 73%, 118 |
|  |  | 2600291610 | NAD(P)-dependent iron-only hydrogenase catalytic subunit | PTH, PTH_2010 | 87%, 1028 |
|  |  | 2600291609 | NAD(P)-dependent iron-only hydrogenase diaphorase component flavoprotein | PTH, PTH_2011 | 83%, 963 |
|  |  | 2600291608 | NADH dehydrogenase subunit E (EC 1.6.5.3) | PTH, PTH_2012 | 82%, 281 |
| **Formate generation & utilization** | Formate ↔ NADH | 2600294272 | Formate dehydrogenase, alpha subunit, archaeal-type | PTH, PTH_2645 | 76%, 863 |
|  |  | 2600291677 | NAD(P)-dependent iron-only hydrogenase diaphorase component flavoprotein | PTH, PTH_2646 | 85%, 635 |
|  |  | 2600291678 | NADH:ubiquinone oxidoreductase 24 kd subunit | PTH, PTH_2647 | 80%, 514 |
|  |  | 2600294269 | NADH-ubiquinone oxidoreductase-G iron-sulfur binding region/Molybdopterin oxidoreductase Fe4S4 domain/4Fe-4S dicluster domain/2Fe-2S iron-sulfur cluster binding domain | PTH, PTH_2648 | 82%, 1021 |
|  |  | 2600294270 | NADH-ubiquinone oxidoreductase-G iron-sulfur binding region/Molybdopterin oxidoreductase Fe4S4 domain/Molybdopterin oxidoreductase/2Fe-2S iron-sulfur cluster binding domain/4Fe-4S dicluster domain | PTH, PTH_2649 | 74%, 264 |
|  | Formate ↔ Menaquinone (MQ); | 2600291823 | Formate dehydrogenase family accessory protein fdhd | PTH, PTH_2610 | 55%, 283 |
|  |  | 2600292822 | Formate dehydrogenase beta subunit (EC 1.2.1.2) | PTH, PTH_1713 | 79%, 421 |
|  |  | 2600294276 | Formate dehydrogenase-N alpha subunit | PTH, PTH_1712 | 68%, 1158 |
|  |  | 2600291681 | Formate dehydrogenase formation protein | PTH, PTH_1711 | 79%, 353 |
|  |  | 2600291682 | Selenocysteine-containing anaerobic dehydrogenase | PTH, PTH_1712 | 68%, 1158 |
|  |  | 2600291683 | Fe-S-cluster-containing hydrogenase components 1 | PTH, PTH_1713 | 79%, 421 |
|  |  | 2600291684 | Transmembrane electron transfer subunit, nrfd | PTH, PTH_1714 | 80%, 623 |
|  |  |  |  |  |  |
| **assimilatory sulfite reduction** | Assimilatory sulfite reduction | 2600293674 | NADPH-dependent flavin oxidoreductase | Desulfosporosinus meridiei | 84%, 441 |
|  |  | 2600293675 | Putative NADPH-dependent hemoprotein | Clostridium acidurici | 51%, 259 |
|  |  | 2600293676 | Anaerobic sulfite reductase, subunit A (asrA) | Clostridium acetobutylicum | 67%, 501 |
|  |  | 2600293677 | Anaerobic sulfite reductase, subunit B (asrB) | Clostridium acetobutylicum | 78%, 451 |
|  |  | 2600293678 | Anaerobic sulfite reductase, subunit C (asrC) | Clostridium acetobutylicum | 83%, 571 |
|  |  | 2600292746 | NADPH-dependent FMN reductase | Desulfotomaculum acetoxidans | 65%, 274 |
|  |  | 2600292787 | NADPH-dependent flavin oxidoreductases | Clostridium sp. | 49%, 292 |
|  |  | 2600292813 | NADPH-dependent FMN reductase | Desulfotomaculum gibsoniae | 67%, 427 |
|  |  | 2600293985 | NADPH-dependent FMN reductase | Desulfotomaculum reducens | 72%, 312 |
|  |  | 2600294448 | NADPH-dependent FMN reductase | Desulfovibrio africanus | 68%, 280 |
|  | Nitrate/sulfonate/bicarbonate transport system | 2600294264 | ABC-type nitrate/sulfonate/bicarbonate transport system, permease component , periplasmic components , and atpase component | Paenibacillus sp. Aloe-11 | 40%, 184 |
|  |  | 2600294265 |  | Syntrophothermus lipocalidus | 33%, 169 |
|  |  | 2600294266 |  | Symbiobacterium thermophilum | 48%, 191 |
|  |  | 2600294338 | ABC-type nitrate/sulfonate/bicarbonate transport system, permease component , periplasmic components , and atpase component | Desulfotomaculum alkaliphilum | 54%, 277 |
|  |  | 2600294339 |  | Desulfotomaculum alkaliphilum | 55%, 250 |
|  |  | 2600294340 |  | Desulfotomaculum alkaliphilum | 55%, 355 |
|  |  | 2600294229 | ABC-type nitrate/sulfonate/bicarbonate transport system, permease component , periplasmic components , and atpase component | Desulfotomaculum reducens | 70%, 402 |
|  |  | 2600294231 |  | Desulfotomaculum reducens | 83%, 283 |
|  |  | 2600294232 |  | Desulfotomaculum reducens | 80%, 407 |

**Table S7 Key KEGG metabolic pathway enzymes encoded in reconstructed genomes**. G3: uncultured *Chloroflexi* T78 clade bacterium; G6: *Brachymonas denitrificans*; G7: *Advenella faeciporci*; G12: *Syntrophus aciditrophicus*; G15: unclutured *Mycobacterium* species; G21: unclutured *Smithella* species.

| **ID** | **Function categories** | **IMG gene ID** | **Functional Annotation Of Protein-Coding Genes** |
| --- | --- | --- | --- |
|  | **G3: uncultured *Chloroflexi* T78 clade bacterium** | | |
| **G3** | **Butyrate degradation** | 2600084384 | 3-Hydroxyacyl-Coa Dehydrogenase (Ec 1.1.1.35) |
|  |  | 2600084739 | 3-Oxoacid Coa-Transferase, A Subunit |
|  |  | 2600085301 | Acetyl-Coa Acetyltransferase |
|  |  | 2600084383 | Enoyl-Coa Hydratase/Carnithine Racemase |
|  |  | 2600084382 | Acetyl-Coa Acetyltransferases |
|  |  | 2600084385 | Butyryl-Coa Dehydrogenase (Ec 1.3.99.2) |
|  |  | 2600084738 | Butyryl-Coa:Acetoacetate Coa-Transferase Beta Subunit (Ec 2.8.3.9) |
|  |  | 2600085300 | Hydroxymethylglutaryl-Coa Synthase, Putative |
|  |  | | |
|  | **Proton reduction**  **(ferredoxin** **↔ H_2_)** | 2600085382 | Nad(P)-Dependent Iron-Only Hydrogenase Catalytic Subunit |
|  |  | 2600085383 | 2fe-2s Iron-Sulfur Cluster Binding Domain |
|  |  | 2600085384 | Nad(P)-Dependent Iron-Only Hydrogenase Diaphorase Component Flavoprotein |
|  |  | 2600085385 | Nad(P)-Dependent Iron-Only Hydrogenase Diaphorase Component Iron-Sulfur Protein |
|  | **Proton translocation** | 2600085557 | Nad(P) Transhydrogenase Beta Subunit |
|  |  | 2600085558 | Nad(P) Transhydrogenase Beta Subunit |
|  |  | 2600085560 | Nad/Nadp Transhydrogenase Alpha Subunit |
|  |  | | |
|  | **Aldehyde and alcohol dehydrogenation**  **(ADH and AdDH)** | 2600084252 | Nad-Dependent Aldehyde Dehydrogenases |
|  |  | 2600084253 | Aldehyde Dehydrogenase Family |
|  |  | 2600085673 | Uncharacterized Oxidoreductases, Fe-Dependent Alcohol Dehydrogenase Family |
|  |  | 2600084573 | Dehydrogenases With Different Specificities (Related To Short-Chain Alcohol Dehydrogenases) |
|  |  | 2600085232 | Dehydrogenases With Different Specificities (Related To Short-Chain Alcohol Dehydrogenases) |
|  |  | 2600085327 | Dehydrogenases With Different Specificities (Related To Short-Chain Alcohol Dehydrogenases) |
|  |  | 2600085334 | Dehydrogenases With Different Specificities (Related To Short-Chain Alcohol Dehydrogenases) |
|  |  | 2600085587 | Dehydrogenases With Different Specificities (Related To Short-Chain Alcohol Dehydrogenases) |
|  |  | 2600085673 | Uncharacterized Oxidoreductases, Fe-Dependent Alcohol Dehydrogenase Family |
|  |  | 2600084633 | Short-Chain Dehydrogenases Of Various Substrate Specificities |
|  |  | 2600085829 | Short-Chain Dehydrogenases Of Various Substrate Specificities |
|  |  | | |
|  | **Glycolysis/Gluconeogenesis** | 2600084531 | 6-Phosphofructokinase (Ec 2.7.1.11) |
|  |  | 2600084324 | Predicted Phosphosugar Isomerases |
|  |  | 2600084568 | Transcriptional Regulator/Sugar Kinase |
|  |  | 2600085718 | Dihydrolipoamide Dehydrogenase |
|  |  | 2600085430 | Enolase (EC 4.2.1.11) |
|  |  | 2600084478 | Fructose-1,6-Bisphosphatase, Class Ii |
|  |  | 2600085597 | Fructose-Bisphosphate Aldolase (Ec 4.1.2.13) |
|  |  | 2600084374 | Glyceraldehyde-3-Phosphate Dehydrogenase (Nad+) (Ec 1.2.1.12) |
|  |  | 2600085094 | Glyceraldehyde-3-Phosphate Dehydrogenase (Nad+) (Ec 1.2.1.12) |
|  |  | 2600084554 | Phosphoenolpyruvate Carboxykinase (Atp) |
|  |  | 2600085779 | Phosphoglucomutase, Alpha-D-Glucose Phosphate-Specific |
|  |  | 2600084375 | Phosphoglycerate Kinase (Ec 2.7.2.3) |
|  |  | 2600084473 | Phosphoglycerate Mutase (Ec 5.4.2.1) |
|  |  | 2600085502 | Pyrophosphate-Dependent Phosphofructokinase (Ec 2.7.1.90) |
|  |  | 2600085521 | Pyruvate Kinase |
|  |  | 2600084571 | Transaldolase (EC 2.2.1.2) |
|  |  | 2600084378 | Triosephosphate Isomerase |
|  | **Pentose phosphate pathway** | 2600084531 | 6-Phosphofructokinase (Ec 2.7.1.11) |
|  |  | 2600084573 | Short-Chain Alcohol Dehydrogenases |
|  |  | 2600084324 | Predicted Phosphosugar Isomerases |
|  |  | 2600084275 | Deoxyribose-Phosphate Aldolase |
|  |  | 2600084478 | Fructose-1,6-Bisphosphatase, Class Ii |
|  |  | 2600085597 | Fructose-Bisphosphate Aldolase (Ec 4.1.2.13) |
|  |  | 2600085779 | Phosphoglucomutase, Alpha-D-Glucose Phosphate-Specific |
|  |  | 2600085502 | Pyrophosphate-Dependent Phosphofructokinase (Ec 2.7.1.90) |
|  |  | 2600084653 | Ribokinase |
|  |  | 2600084964 | Ribose 5-Phosphate Isomerase B |
|  |  | 2600084961 | Ribose-Phosphate Pyrophosphokinase |
|  |  | 2600085463 | Ribulose-5-Phosphate 3-Epimerase (Ec 5.1.3.1) |
|  |  | 2600084571 | Transaldolase (EC 2.2.1.2) |
|  |  | 2600084572 | Transketolase, Bacterial And Yeast |
| **G6: *Brachymonas Denitrificans*** | | | |
| **G6** | **Uptake of extracellular Nitrate/nitrite** | 2600286161 | Nitrate/Nitrite Transporter (Nrt) |
|  | **Nitrate/nitrite denitrification**  **(Nitrate↔Nitrite)** | 2600286164 | Respiratory Nitrate Reductase Alpha Subunit Apoprotein (Narg) |
|  |  | 2600286165 | Respiratory Nitrate Reductase Beta Subunit (Narh) |
|  |  | 2600286166 | Respiratory Nitrate Reductase Chaperone Narj (Narj) |
|  |  | 2600286167 | Respiratory Nitrate Reductase Gamma Subunit (Nari) |
|  | **Nitrate/nitrite denitrification (Nitrite→Nitric oxide)** | 2600284316 | Nitrite Reductase (NO-Forming) |
|  |  | 2600284318 | Nitrite Reductase (NO-Forming) |
|  | **Nitrate/nitrite denitrification (Nitric oxide→Nitrous oxide)** | 2600284305 | Nitric Oxide Reductase (Norb) |
|  |  | 2600284306 | Nitric Oxide Reductase (Norc) |
|  | **Nitrate/nitrite denitrification (Nitrous oxide****→Nitrogen)** | 2600285578 | Nitrous Oxide Reductase Apoprotein |
|  | **Nitroalkane→Nitrite** | 2600284357 | Dioxygenases Related To 2-Nitropropane Dioxygenase |
|  |  | 2600284653 | Dioxygenases Related To 2-Nitropropane Dioxygenase |
|  |  | 2600286100 | Dioxygenases Related To 2-Nitropropane Dioxygenase |
|  |  | | |
| **G6** | **Electron transport complex** | 2600284279 | Electron Transport Complex, Rnfabcdge Type |
|  |  | 2600284280 | Electron Transport Complex, Rnfabcdge Type |
|  | **Oxygen tolerance** | 2600286150 | Cbb3-Type Cytochrome Oxidase, Cytochrome C Subunit |
|  |  | 2600286149 | Cbb3-Type Cytochrome Oxidase, Subunit 1 |
|  |  | 2600285331 | Cbb3-Type Cytochrome Oxidase, Subunit 3 |
|  | **Oxidative tolerance** | 2600285329 | Cytochrome C Oxidase, Cbb3-Type, Subunit I |
|  |  | 2600285332 | Cytochrome C Oxidase, Cbb3-Type, Subunit Iii |
|  |  | 2600283690 | Cytochrome D Oxidase, Subunit Ii (Cydb) |
|  |  | 2600284856 | Uncharacterized Peroxidase-Related Enzyme |
|  |  | 2600285587 | Alkylhydroperoxidase Ahpd Family Core Domain |
|  |  | 2600285995 | Alkylhydroperoxidase Ahpd Family Core Domain |
|  |  | 2600283735 | Cu/Zn Superoxide Dismutase |
|  |  | 2600284485 | Superoxide Dismutase |
|  |  | | |
| **G6** | **Propionate degradation (I):**  **MMC pathway** | 2600284791 | Propionyl-Coa Synthetase (EC 6.2.1.17) |
|  |  | 2600284271 | Acetyl-Coa Carboxylase, Beta Subunit |
|  |  | 2600284272 | Acetyl-Coa Carboxylase, Alpha Subunit |
|  |  | 2600284278 | Methylmalonyl-Coa Epimerase |
|  |  | 2600284269 | Methylmalonyl-Coa Mutase N-Terminal Domain |
|  |  | 2600284270 | Methylmalonyl-Coa Mutase C-Terminal Domain |
|  |  | 2600285518 | Succinyl-Coa Synthetase, Beta Subunit |
|  |  | 2600285519 | Succinyl-Coa Synthetase, Alpha Subunit |
|  | **Propionate degradation (II):**  **Reductive carboxylation** | 2600284791 | Propionyl-Coa Synthetase (EC 6.2.1.17) |
|  |  | 2600284069 | Methylmalonic Acid Semialdehyde Dehydrogenase (EC 1.2.1.27; 1.2.1.18 ) |
|  |  | 2600284269 | Methylmalonyl-Coa Mutase N-Terminal Domain |
|  |  | 2600284270 | Methylmalonyl-Coa Mutase C-Terminal Domain |
|  | **Propionate degradation (III):**  **Acryloyl-CoA pathway** | 2600284537 | Acyl-Coa Dehydrogenases |
|  |  | 2600284538 | Acyl-Coa Dehydrogenase, N-Terminal Domain |
|  |  | 2600284147 | Enoyl-Coa Hydratase (EC 4.2.1.17) |
|  |  | 2600285413 | Enoyl-Coa Hydratase (EC 4.2.1.17) |
|  |  | 2600283850 | Malonyl-Coa Decarboxylase (MCD) |
|  |  | 2600285112 | Acetyl-Coa Carboxylase, Carboxyl Transferase, Beta Subunit (EC 6.4.1.2) |
|  |  | 2600285424 | Acetyl-Coa Carboxylase, Biotin Carboxyl Carrier Protein (EC 6.4.1.2) |
|  |  | 2600285425 | Acetyl-Coa Carboxylase, Biotin Carboxylase Subunit (EC 6.4.1.2) |
|  |  | 2600285820 | Acetyl-Coa Carboxylase, Carboxyl Transferase, Alpha Subunit (EC 6.4.1.2) |
|  |  | | |
| **G6** | **Lactate→Puryrate** | 2600284990 | D-Lactate Dehydrogenase (Cytochrome) |
|  | **Benzoate→Catechol**  **(oxic/anoxic conditions)** | 2600284076 | Dihydroxycyclohexadiene Carboxylate Dehydrogenase |
|  |  | 2600284077 | Benzoate/Toluate 1,2-Dioxygenase Reductase Subunit |
|  |  | 2600284078 | Benzoate 1,2-Dioxygenase, Small Subunit |
|  | **Phenol→Catechol**  **(oxic/anoxic conditions)** | 2600286109 | Phenol 2-Monooxygenase P0 Subunit (EC 1.14.13.7) |
|  |  | | |
| **G6** | **Pyruvate metabolism** | 2600286106 | 2-Isopropylmalate Synthase (EC 2.3.3.13) |
|  |  | 2600284990 | FAD/FMN-Containing Dehydrogenases |
|  |  | 2600284278 | Glyoxalase/Bleomycin Resistance Protein/Dioxygenase Superfamily |
|  |  | 2600285567 | L-Lactate Dehydrogenase (FMN-Dependent) And Related Alpha-Hydroxy Acid Dehydrogenases |
|  |  | 2600285208 | Lactoylglutathione Lyase And Related Lyases |
|  |  | 2600284983 | Malic Enzyme |
|  |  | 2600285063 | Malic Enzyme |
|  |  | 2600285226 | Phosphoenolpyruvate Carboxykinase (GTP) |
|  |  | 2600283915 | Phosphotransacetylase |
|  |  | 2600284667 | Pyruvate Kinase, Barrel Domain |
|  |  | 2600283916 | Acetate Kinase |
|  |  | 2600284529 | Acetyl-Coa Acetyltransferases |
|  |  | 2600284915 | Acetyl-Coa Acetyltransferases |
|  |  | 2600285424 | Acetyl-Coa Carboxylase, Biotin Carboxyl Carrier Protein |
|  |  | 2600285425 | Acetyl-Coa Carboxylase, Biotin Carboxylase Subunit |
|  |  | 2600285820 | Acetyl-Coa Carboxylase, Carboxyl Transferase, Alpha Subunit |
|  |  | 2600285112 | Acetyl-Coa Carboxylase, Carboxyl Transferase, Beta Subunit |
|  |  | 2600284788 | Dihydrolipoamide Dehydrogenase |
|  |  | 2600284340 | Fumarase, Class I, Homodimeric (EC 4.2.1.2) |
|  |  | 2600285509 | Fumarase, Class II (EC 4.2.1.2) |
|  |  | 2600284183 | Hydroxyacylglutathione Hydrolase |
|  |  | 2600283804 | Lactoylglutathione Lyase |
|  |  | 2600285149 | Malate Dehydrogenase (NAD) (EC 1.1.1.37) |
|  |  | 2600284646 | Malate Synthase (EC 2.3.3.9) |
|  |  | 2600284718 | Phosphoenolpyruvate Synthase (EC 2.7.9.2) |
|  |  | 2600284786 | Pyruvate Dehydrogenase E1 Component, Homodimeric Type |
|  |  | 2600284787 | Pyruvate Dehydrogenase Complex Dihydrolipoamide Acetyltransferase, Long Form |
|  |  | 2600285043 | Pyruvate Kinase |
|  |  | 2600285979 | Pyruvate Phosphate Dikinase (EC 2.7.9.1) |
|  |  | 2600285091 | Succinate Coa Transferase |
|  |  | 2600285343 | Succinate Dehydrogenase And Fumarate Reductase Iron-Sulfur Protein |
|  |  | 2600285913 | Succinate Dehydrogenase Subunit C (EC 1.3.5.1) |
| **G7: *Advenella Faeciporci*** | | | |
| **G7** | **Nitrite denitrification (Nitrite→Nitric oxide)** | 2600288241 | Dissimilatory Nitrite Reductase (NO-Forming), Copper Type Apoprotein |
|  | **Nitrite denitrification**  **(Nitric oxide→Nitrous oxide)** | 2600287119 | Nitric Oxide Reductase Large Subunit |
|  |  | 2600287336 | Nitric Oxide Reductase, Norz Apoprotein |
|  | **Nitrite denitrification**  **(Nitrous oxide→Nitrogen)** | 2600288845 | NADP Nitrous Oxide-Forming Nitric Oxide Reductase |
|  | **Nitroalkane→Nitrite** | 2600287839 | Dioxygenases Related To 2-Nitropropane Dioxygenase |
|  |  | 2600288216 | Dioxygenases Related To 2-Nitropropane Dioxygenase |
|  |  | 2600288592 | Dioxygenases Related To 2-Nitropropane Dioxygenase |
|  | **Oxygen tolerance** | 2600287806 | Cytochrome C Oxidase, Cbb3-Type, Subunit I |
|  |  | 2600287807 | Cytochrome C Oxidase, Cbb3-Type, Subunit Ii |
|  |  | 2600287808 | Cytochrome C Oxidase, Cbb3-Type, Subunit Iii |
|  | **Oxidative tolerance** | 2600286462 | Superoxide Dismutase |
|  |  | 2600286247 | Cytochrome C Peroxidase |
|  |  | 2600287073 | Glutathione Peroxidase |
|  |  | 2600287701 | Uncharacterized Peroxidase-Related Enzyme |
|  | **Electron transfer complex** | 2600288282 | Electron Transport Complex, Rnfabcdge Type, B Subunit |
|  | **Propionate metabolism**  **(Acryloyl-CoA pathway)** | 2600286251 | Acyl-Coa Dehydrogenases |
|  |  | 2600287230 | Acyl-Coa Dehydrogenases |
|  |  | 2600287436 | Acyl-Coa Dehydrogenases |
|  |  | 2600288756 | Acyl-Coa Dehydrogenases |
|  |  | 2600286899 | Enoyl-Coa Hydratase/Carnithine Racemase |
|  |  | 2600288135 | Enoyl-Coa Hydratase/Carnithine Racemase |
|  |  | 2600286541 | Acetyl-Coa Carboxylase, Biotin Carboxyl Carrier Protein |
|  |  | 2600286542 | Acetyl-Coa Carboxylase, Biotin Carboxylase Subunit |
|  |  | 2600287456 | Acetyl-Coa Carboxylase, Carboxyl Transferase, Alpha Subunit |
|  |  | 2600288292 | Acetyl-Coa Carboxylase, Carboxyl Transferase, Beta Subunit |
|  | **Lactate→Puryrate** | 2600289021 | D-Lactate Dehydrogenase (Cytochrome) |
|  |  | 2600287653 | L-Lactate Dehydrogenase (FMN-Dependent) And Related Alpha-Hydroxy Acid Dehydrogenases |
|  |  | 2600287851 | L-Lactate Dehydrogenase (FMN-Dependent) And Related Alpha-Hydroxy Acid Dehydrogenases |
|  | **Catechol** **degradation to Acetyl-CoA**  **(oxic/anoxic conditions)** | 2600288930 | Catechol 1,2-Dioxygenase, Proteobacterial (Cata) |
|  |  | 2600288929 | Muconate And Chloromuconate Cycloisomerases (Catb) |
|  |  | 2600288932 | Muconolactone Delta-Isomerase (Catc) |
|  |  | 2600288931 | 3-Oxoadipate Enol-Lactonase (Pcad) |
|  |  | 2600287468 | 3-Oxoacid Coa-Transferase, A Subunit (Pcai) |
|  |  | 2600287469 | 3-Oxoacid Coa-Transferase, B Subunit (Pcaj) |
|  |  | 2600288222 | 3-Oxoacid Coa-Transferase, A Subunit (Pcai) |
|  |  | 2600288223 | 3-Oxoacid Coa-Transferase, B Subunit (Pcaj) |
|  | **4-OHB degradation**  **(oxic/anoxic conditions)** | 2600288642 | 4-Hydroxybenzoate 3-Monooxygenase (Poba; EC 1.14.13.2) |
|  |  | 2600288633 | Protocatechuate 4,5-Dioxygenase Alpha Subunit (Liga; EC 1.13.11.8) |
|  |  | 2600288634 | Catalytic Ligb Subunit Of Aromatic Ring-Opening Dioxygenases (Ligb; EC 1.13.11.8) |
|  |  | 2600288790 | Protocatechuate 4,5-Dioxygenase Beta Subunit (Ligb ; EC 1.13.11.8) |
|  |  | 2600288635 | 2-Hydroxy-4-Carboxymuconate Semialdehyde Hemiacetal Dehydrogenase (Ligc; EC 1.1.132) |
|  |  | 2600288632 | Predicted Metal-Dependent Hydrolase Of The TIM-Barrel Fold (Ligi; EC 3.1.1.57) |
|  |  | 2600288628 | Putative 4-Oxalomesaconate Tautomerase (Gald; EC 5.3.2.8) |
|  |  | 2600288630 | 4-Oxalomesaconate Hydratase (Ligj; Galc; EC 4.2.1.83) |
|  |  | 2600288631 | 4-Carboxy-4-Hydroxy-2-Oxoadipate Aldolase (Ligk; Galc; EC 4.1.3.17) |
|  | **Pyruvate metabolism** | 2600287925 | 2-Isopropylmalate Synthase, Yeast Type |
|  |  | 2600287322 | Acyl-Coenzyme A Synthetases/Amp-(Fatty) Acid Ligases |
|  |  | 2600289021 | Fad/Fmn-Containing Dehydrogenases |
|  |  | 2600287653 | L-Lactate Dehydrogenase (Fmn-Dependent) And Related Alpha-Hydroxy Acid Dehydrogenases |
|  |  | 2600287851 | L-Lactate Dehydrogenase (Fmn-Dependent) And Related Alpha-Hydroxy Acid Dehydrogenases |
|  |  | 2600288623 | Lactoylglutathione Lyase And Related Lyases |
|  |  | 2600287397 | Malic Enzyme |
|  |  | 2600288049 | Malic Enzyme |
|  |  | 2600288579 | Nad-Dependent Aldehyde Dehydrogenases |
|  |  | 2600288645 | Nad-Dependent Aldehyde Dehydrogenases |
|  |  | 2600286971 | Phosphoenolpyruvate Carboxylase, Type 1 (Ec 4.1.1.31) |
|  |  | 2600288069 | Phosphoglycerate Dehydrogenase And Related Dehydrogenases |
|  |  | 2600287985 | Phosphotransacetylase |
|  |  | 2600289036 | Pyruvate/2-Oxoglutarate Dehydrogenase Complex, Dihydrolipoamide Dehydrogenase (E3) |
|  |  | 2600287659 | Thiamine Pyrophosphate-Requiring Enzymes |
|  |  | 2600287915 | Acetaldehyde Dehydrogenase (Ec 1.2.1.10) |
|  |  | 2600287986 | Acetate Kinase |
|  |  | 2600288902 | Acetate--Coa Ligase |
|  |  | 2600286253 | Acetyl-Coa Acetyltransferases |
|  |  | 2600287106 | Acetyl-Coa Acetyltransferases |
|  |  | 2600287939 | Acetyl-Coa Acetyltransferases |
|  |  | 2600288330 | Acetyl-Coa Acetyltransferases |
|  |  | 2600286541 | Acetyl-Coa Carboxylase, Biotin Carboxyl Carrier Protein |
|  |  | 2600286542 | Acetyl-Coa Carboxylase, Biotin Carboxylase Subunit |
|  |  | 2600287456 | Acetyl-Coa Carboxylase, Carboxyl Transferase, Alpha Subunit |
|  |  | 2600288292 | Acetyl-Coa Carboxylase, Carboxyl Transferase, Beta Subunit |
|  |  | 2600287028 | Dihydrolipoamide Dehydrogenase |
|  |  | 2600287394 | Dihydrolipoamide Dehydrogenase |
|  |  | 2600288136 | Fumarase, Class I, Homodimeric (Ec 4.2.1.2) |
|  |  | 2600288766 | Fumarase, Class Ii (Ec 4.2.1.2) |
|  |  | 2600288013 | Hydroxyacylglutathione Hydrolase |
|  |  | 2600287447 | Lactoylglutathione Lyase |
|  |  | 2600288129 | Lactoylglutathione Lyase |
|  |  | 2600287406 | Malate Dehydrogenase |
|  |  | 2600287731 | Malate Synthase G |
|  |  | 2600287660 | Malate:Quinone-Oxidoreductase |
|  |  | 2600287055 | Phosphoenolpyruvate Synthase (Ec 2.7.9.2) |
|  |  | 2600287026 | Pyruvate Dehydrogenase E1 Component, Homodimeric Type |
|  |  | 2600287135 | Pyruvate Dehydrogenase E1 Component, Homodimeric Type |
|  |  | 2600287027 | Pyruvate Dehydrogenase Complex Dihydrolipoamide Acetyltransferase |
|  |  | 2600288125 | Pyruvate Kinase (Ec 2.7.1.40) |
|  |  | 2600288941 | Succinate Coa Transferase |
|  | | | |
| **G12: *Syntrophus Aciditrophicus*** | | | |
| **G12** | **Butyrate metabolism** | 2600289692 | 2-Oxoacid:Acceptor Oxidoreductase, Delta Subunit, Pyruvate/2-Ketoisovalerate Family |
|  |  | 2600289693 | 2-Oxoacid:Acceptor Oxidoreductase, Gamma Subunit, Pyruvate/2-Ketoisovalerate Family |
|  |  | 2600289340 | 3-Hydroxyacyl-Coa Dehydrogenase |
|  |  | 2600291280 | 4-Aminobutyrate Aminotransferase And Related Aminotransferases |
|  |  | 2600290259 | Acetyl-Coa Acetyltransferase |
|  |  | 2600289339 | Enoyl-Coa Hydratase/Carnithine Racemase |
|  |  | 2600289691 | Pyruvate:Ferredoxin Oxidoreductase And Related 2-Oxoacid:Ferredoxin Oxidoreductases |
|  |  | 2600289181 | Thiamine Pyrophosphate Enzyme, N-Terminal Tpp Binding Domain |
|  |  | 2600290287 | Acetolactate Synthase, Large Subunit (Ec 2.2.1.6) |
|  |  | 2600290289 | Acetolactate Synthase, Large Subunit (Ec 2.2.1.6) |
|  |  | 2600290286 | Acetolactate Synthase, Small Subunit (Ec 2.2.1.6) |
|  |  | 2600290288 | Acetolactate Synthase, Small Subunit (Ec 2.2.1.6) |
|  |  | 2600290685 | Acetyl-Coa Acetyltransferase (Ec 2.3.1.9) |
|  |  | 2600291354 | Phosphate Butyryltransferase (Ec 2.3.1.19) |
|  |  | 2600291355 | Phosphate Butyryltransferase (Ec 2.3.1.19) |
|  |  | 2600289690 | Pyruvate Ferredoxin Oxidoreductase, Beta Subunit (Ec 1.2.7.1) |
|  |  | 2600290057 | Vinylacetyl-Coa Delta-Isomerase/4-Hydroxybutyryl-Coa Dehydratase (Ec 4.2.1.-) |
|  |  | | |
| **G12** | **Propionate metabolism**  **(MMC pathway)** | 2600289692 | 2-Oxoacid:Acceptor Oxidoreductase, Delta Subunit, Pyruvate/2-Ketoisovalerate Family |
|  |  | 2600289693 | 2-Oxoacid:Acceptor Oxidoreductase, Gamma Subunit, Pyruvate/2-Ketoisovalerate Family |
|  |  | 2600291280 | 4-Aminobutyrate Aminotransferase And Related Aminotransferases |
|  |  | 2600290860 | Atp-Grasp Domain |
|  |  | 2600290259 | Acetyl-Coa Acetyltransferase |
|  |  | 2600289299 | Acetyl-Coa Carboxylase, Carboxyltransferase Component (Subunits Alpha And Beta) |
|  |  | 2600289943 | Acyl-Coa Dehydrogenases |
|  |  | 2600290196 | Acyl-Coa Dehydrogenases |
|  |  | 2600290277 | Acyl-Coa Dehydrogenases |
|  |  | 2600290866 | Biotin-Requiring Enzyme |
|  |  | 2600291036 | Nad-Dependent Aldehyde Dehydrogenases |
|  |  | 2600289691 | Pyruvate:Ferredoxin Oxidoreductase And Related 2-Oxoacid:Ferredoxin Oxidoreductases |
|  |  | 2600291388 | Threonine Dehydrogenase And Related Zn-Dependent Dehydrogenases |
|  |  | 2600290685 | Acetyl-Coa Acetyltransferase (Ec 2.3.1.9) |
|  |  | 2600290678 | Acetyl-Coenzyme A Synthetase (Ec 6.2.1.1) |
|  |  | 2600291288 | Acetyl-Coenzyme A Synthetase (Ec 6.2.1.1) |
|  |  | 2600289385 | Methylmalonyl-Coa Epimerase (Ec 5.1.99.1) |
|  |  | 2600289690 | Pyruvate Ferredoxin Oxidoreductase, Beta Subunit (Ec 1.2.7.1) |
|  |  | 2600291453 | Succinyl-Coa Synthetase (Adp-Forming) Alpha Subunit (Ec 6.2.1.5) |
|  |  | 2600289915 | Succinyl-Coa Synthetase (Adp-Forming) Beta Subunit (Ec 6.2.1.5) |
|  |  | 2600291454 | Succinyl-Coa Synthetase (Adp-Forming) Beta Subunit (Ec 6.2.1.5) |
| **G15: Unclutured *Mycobacterium* Species** | | | |
| **G15** | **Sulfate→3'-phosphoadenosine 5'-phosphosulfate (PAPS)** | 2600298342 | Adenylylsulfate Kinase (EC 2.7.1.25)/Sulfate Adenylyltransferase(EC 2.7.7.4) (Cysn; Cysc) |
|  |  | 2600298343 | Sulfate Adenylyltransferase Subunit 2 (EC 2.7.7.4) (Cysd) |
|  | **PAPS→Sulfite** | 2600298068 | Phosophoadenylyl-Sulfate Reductase (Thioredoxin) (Cysh) |
|  | **Sulfite→Sulfide** | 2600298069 | Sulfite Reductase, Beta Subunit (Hemoprotein) |
|  | **Sulfate transprotation** | 2600298066 | Sulfate Abc Transporter, Atp-Binding Protein |
|  |  | 2600298064 | Sulfate Abc Transporter, Permease Protein Cyst |
|  |  | 2600298065 | Sulfate Abc Transporter, Permease Protein Cysw |
|  | **Other enzymes related to sulfur metabolism** | 2600294630 | 3’-Phosphoadenosine 5’-Phosphosulfate (Paps) 3’-Phosphatase |
|  |  | 2600298341 | 3’-Phosphoadenosine 5’-Phosphosulfate (Paps) 3’-Phosphatase |
|  |  | 2600294889 | Cysteine Synthase |
|  |  | 2600297034 | Cysteine Synthase |
|  |  | 2600296213 | O-Succinylhomoserine Sulfhydrylase |
|  |  | 2600297310 | Rhodanese-Related Sulfurtransferase |
|  |  | 2600299490 | Rhodanese-Related Sulfurtransferase |
|  |  | 2600298798 | Uncharacterized Nad(Fad)-Dependent Dehydrogenases |
|  |  | 2600298848 | Cystathionine Gamma-Lyase (Ec 4.4.1.1) |
|  |  | 2600295403 | Cysteine Synthase A |
|  |  | 2600296820 | Dimethyl Sulfone Monooxygenase Sfng |
|  |  | 2600295404 | Serine O-Acetyltransferase |
|  |  | 2600298063 | Sulfate/Thiosulfate-Binding Protein |
|  |  | 2600296474 | Thiosulfate Sulfurtransferase (Ec 2.8.1.1) |
|  |  | | |
| **G15** | **Naphthalene biodegradation** | 2600298319 | Alcohol Dehydrogenase Groes-Like Domain/Zinc-Binding Dehydrogenase |
|  |  | 2600295933 | Nad-Dependent Aldehyde Dehydrogenases |
|  |  | 2600295095 | Ndma-Dependent Alcohol Dehydrogenase, Rxyl_3153 Family |
|  |  | 2600295174 | Ndma-Dependent Alcohol Dehydrogenase, Rxyl_3153 Family |
|  |  | 2600299105 | Ndma-Dependent Alcohol Dehydrogenase, Rxyl_3153 Family |
|  |  | 2600296706 | Predicted Flavoprotein Involved In K+ Transport |
|  |  | 2600298187 | Zn-Dependent Alcohol Dehydrogenases |
|  |  | 2600298320 | Acetaldehyde Dehydrogenase (Ec 1.2.1.10)/Alcohol Dehydrogenase Adhe (Ec 1.1.1.1) |
|  | **Dichloropropene biodegradation** | 2600295690 | Putative Haloalkane Dehalogenase |
|  |  | 2600298558 | Putative Haloalkane Dehalogenase |
|  |  | 2600295028 | NAD-Dependent Aldehyde Dehydrogenases |
|  |  | 2600295181 | Aldehyde Dehydrogenase, Rv0768 Family |
|  |  | 2600296704 | Aldehyde Dehydrogenase, Rv0768 Family |
|  |  | 2600296994 | NAD-Dependent Aldehyde Dehydrogenases |
|  |  | 2600298807 | NAD-Dependent Aldehyde Dehydrogenases |
|  |  | 2600296659 | Putative Haloacetate Dehalogenase |
|  |  | 2600299351 | 2-Haloalkanoic Acid Dehalogenase, Type II |
|  | **Phenanthrene biodegradation** | 2600296706 | E1.14.13.- (Predicted Flavoprotein Involved In K+ Transport) |
|  |  | 2600295785 | Methyltransferase-Like Protein (METTL6;EC:2.1.1.-) |
|  |  | 2600296962 | Methyltransferase-Like Protein (METTL6;EC:2.1.1.-) |
|  |  | 2600297400 | Methyltransferase-Like Protein (METTL6;EC:2.1.1.-) |
|  |  | 2600298226 | Methyltransferase-Like Protein (METTL6;EC:2.1.1.-) |
|  |  | | |
| **G15** | **Propionate biodegradation:**  **(I) MMC pathway and (II) Reductive carboxylation pathway** | 2600294997 | 3-Ketoacyl-Coa Thiolase (Ec 2.3.1.16) |
|  |  | 2600298999 | 4-Aminobutyrate Aminotransferase, Prokaryotic Type |
|  |  | 2600295130 | Acetyl-Coa Acetyltransferase |
|  |  | 2600296822 | Acetyl-Coa Acetyltransferase |
|  |  | 2600296872 | Acetyl-Coa Acetyltransferase |
|  |  | 2600299487 | Acetyl-Coa Carboxylase, Carboxyltransferase Component (Subunits Alpha And Beta) |
|  |  | 2600296810 | Acetyl/Propionyl-Coa Carboxylase, Alpha Subunit |
|  |  | 2600294968 | Acyl-Coa Dehydrogenase, C-Terminal Domain |
|  |  | 2600294957 | Acyl-Coa Dehydrogenases |
|  |  | 2600296263 | Acyl-Coenzyme A Synthetases/Amp-(Fatty) Acid Ligases |
|  |  | 2600296411 | Acyl-Coenzyme A Synthetases/Amp-(Fatty) Acid Ligases |
|  |  | 2600294961 | Enoyl-Coa Hydratase/Carnithine Racemase |
|  |  | 2600295102 | Enoyl-Coa Hydratase/Carnithine Racemase |
|  |  | 2600295028 | Nad-Dependent Aldehyde Dehydrogenases |
|  |  | 2600296994 | Nad-Dependent Aldehyde Dehydrogenases |
|  |  | 2600298807 | Nad-Dependent Aldehyde Dehydrogenases |
|  |  | 2600296198 | Acetate Kinase |
|  |  | 2600296431 | Acetyl-Coa Acetyltransferases |
|  |  | 2600298842 | Acetyl-Coa Acetyltransferases |
|  |  | 2600294871 | Acetyl-Coenzyme A Synthetase (Ec 6.2.1.1) |
|  |  | 2600295181 | Aldehyde Dehydrogenase, Rv0768 Family |
|  |  | 2600296704 | Aldehyde Dehydrogenase, Rv0768 Family |
|  |  | 2600295312 | Glycerol Dehydratase, Cobalamin-Independent, Large Subunit (Ec 4.2.1.30) |
|  |  | 2600297904 | Hypothetical Protein |
|  |  | 2600298670 | Malate Dehydrogenase (Nad) (Ec 1.1.1.37) |
|  |  | 2600295483 | Methylmalonate-Semialdehyde Dehydrogenase [Acylating] (Ec 1.2.1.27) |
|  |  | 2600297277 | Methylmalonic Acid Semialdehyde Dehydrogenase |
|  |  | 2600295968 | Methylmalonyl-Coa Epimerase (Ec 5.1.99.1) |
|  |  | 2600295930 | Methylmalonyl-Coa Mutase (Ec 5.4.99.2) |
|  |  | 2600295929 | Methylmalonyl-Coa Mutase C-Terminal Domain |
|  |  | 2600296118 | Methylmalonyl-Coa Mutase C-Terminal Domain/Methylmalonyl-Coa Mutase N-Terminal Domain |
|  |  | 2600296199 | Phosphate Acetyltransferase |
|  |  | 2600296849 | Propionyl-Coa Synthetase (Ec 6.2.1.17) |
|  |  | 2600295137 | Short Chain Enoyl-Coa Hydratase (Ec 4.2.1.17) |
|  |  | 2600298839 | Short Chain Enoyl-Coa Hydratase (Ec 4.2.1.17) |
|  |  | 2600296823 | Succinyl-Coa Synthetase (Adp-Forming) Alpha Subunit (Ec 6.2.1.5) |
|  |  | 2600296824 | Succinyl-Coa Synthetase, Beta Subunit |
|  |  | | |
| **G15** | **Butyrate/Butanol biodegradation** | 2600298747 | 3-Hydroxyacyl-Coa Dehydrogenase |
|  |  | 2600294997 | 3-Ketoacyl-Coa Thiolase (Ec 2.3.1.16) |
|  |  | 2600298999 | 4-Aminobutyrate Aminotransferase, Prokaryotic Type |
|  |  | 2600295130 | Acetyl-Coa Acetyltransferase |
|  |  | 2600296822 | Acetyl-Coa Acetyltransferase |
|  |  | 2600296872 | Acetyl-Coa Acetyltransferase |
|  |  | 2600299200 | Acyl-Coa Dehydrogenases |
|  |  | 2600295030 | Aldehyde Dehydrogenase Family |
|  |  | 2600295946 | Dehydrogenases Related To Short-Chain Alcohol Dehydrogenases |
|  |  | 2600294961 | Enoyl-Coa Hydratase/Carnithine Racemase |
|  |  | 2600295102 | Enoyl-Coa Hydratase/Carnithine Racemase |
|  |  | 2600296359 | Hmgl-Like |
|  |  | 2600299093 | Hmgl-Like |
|  |  | 2600294652 | Maleate Cis-Trans Isomerase |
|  |  | 2600296832 | Nad-Dependent Aldehyde Dehydrogenases |
|  |  | 2600297314 | Predicted Dehydrogenase |
|  |  | 2600295004 | Succinate Dehydrogenase/Fumarate Reductase, Flavoprotein Subunit |
|  |  | 2600298145 | Thiamine Pyrophosphate Enzyme, C-Terminal Tpp Binding Domain |
|  |  | 2600297086 | Thiamine Pyrophosphate-Requiring Enzymes |
|  |  | 2600295119 | Acetaldehyde Dehydrogenase (Ec 1.2.1.10) |
|  |  | 2600298320 | Acetaldehyde Dehydrogenase (Ec 1.2.1.10)/Alcohol Dehydrogenase Adhe (Ec 1.1.1.1) |
|  |  | 2600295931 | Acetoacetyl-Coa Synthase |
|  |  | 2600294946 | Acetolactate Synthase, Small Subunit (Ec 2.2.1.6) |
|  |  | 2600295094 | Acetyl-Coa Acetyltransferases |
|  |  | 2600295108 | Acetyl-Coa Acetyltransferases |
|  |  | 2600299633 | Glutamate Decarboxylase (Ec 4.1.1.15) |
|  |  | 2600295312 | Glycerol Dehydratase, Cobalamin-Independent, Large Subunit (Ec 4.2.1.30) |
|  |  | 2600297904 | Hypothetical Protein |
|  |  | 2600295137 | Short Chain Enoyl-Coa Hydratase (Ec 4.2.1.17) |
|  |  | 2600298839 | Short Chain Enoyl-Coa Hydratase (Ec 4.2.1.17) |
|  |  | 2600295003 | Succinate Dehydrogenase And Fumarate Reductase Iron-Sulfur Protein |
|  |  | 2600297588 | Succinate Dehydrogenase And Fumarate Reductase Iron-Sulfur Protein |
|  |  | 2600297586 | Succinate Dehydrogenase Subunit A (Ec 1.3.5.1) |
|  |  | 2600298776 | Succinate Dehydrogenase Subunit D (Ec 1.3.5.1) |
|  |  | | |
| **G15** | **Fatty acid degradation** | 2600294997 | 3-Ketoacyl-Coa Thiolase (Ec 2.3.1.16) |
|  |  | 2600295130 | Acetyl-Coa Acetyltransferase |
|  |  | 2600296779 | Acetyl-Coa Acetyltransferase |
|  |  | 2600294968 | Acyl-Coa Dehydrogenase, C-Terminal Domain |
|  |  | 2600294957 | Acyl-Coa Dehydrogenases |
|  |  | 2600294969 | Acyl-Coa Dehydrogenases |
|  |  | 2600294761 | Acyl-Coenzyme A Oxidase (Ec 1.3.3.6) |
|  |  | 2600298319 | Alcohol Dehydrogenase Groes-Like Domain/Zinc-Binding Dehydrogenase |
|  |  | 2600294961 | Enoyl-Coa Hydratase/Carnithine Racemase |
|  |  | 2600295102 | Enoyl-Coa Hydratase/Carnithine Racemase |
|  |  | 2600298519 | Fatty Acid Desaturase |
|  |  | 2600297768 | Long-Chain Acyl-Coa Synthetases (Amp-Forming) |
|  |  | 2600299049 | Long-Chain Acyl-Coa Synthetases (Amp-Forming) |
|  |  | 2600295028 | Nad-Dependent Aldehyde Dehydrogenases |
|  |  | 2600296994 | Nad-Dependent Aldehyde Dehydrogenases |
|  |  | 2600298807 | Nad-Dependent Aldehyde Dehydrogenases |
|  |  | 2600295095 | Ndma-Dependent Alcohol Dehydrogenase, Rxyl_3153 Family |
|  |  | 2600295174 | Ndma-Dependent Alcohol Dehydrogenase, Rxyl_3153 Family |
|  |  | 2600299105 | Ndma-Dependent Alcohol Dehydrogenase, Rxyl_3153 Family |
|  |  | 2600296908 | Pyridine Nucleotide-Disulphide Oxidoreductase |
|  |  | 2600299473 | Uncharacterized Nad(Fad)-Dependent Dehydrogenases |
|  |  | 2600298187 | Zn-Dependent Alcohol Dehydrogenases |
|  |  | 2600298320 | Acetaldehyde Dehydrogenase/Alcohol Dehydrogenase Adhe |
|  |  | 2600295094 | Acetyl-Coa Acetyltransferases |
|  |  | 2600295108 | Acetyl-Coa Acetyltransferases |
|  |  | 2600295181 | Aldehyde Dehydrogenase, Rv0768 Family |
|  |  | 2600296704 | Aldehyde Dehydrogenase, Rv0768 Family |
|  |  | 2600297904 | Hypothetical Protein |
|  |  | 2600295137 | Short Chain Enoyl-Coa Hydratase (Ec 4.2.1.17) |
|  |  | 2600298839 | Short Chain Enoyl-Coa Hydratase (Ec 4.2.1.17) |
|  |  | | |
| **G15** | **Starch and sucrose metabolism** | 2600299568 | 4-Alpha-Glucanotransferase |
|  |  | 2600296289 | Beta-Glucosidase-Related Glycosidases |
|  |  | 2600296624 | Glycosidases |
|  |  | 2600294836 | Glycosyl Hydrolases Family 6 |
|  |  | 2600299024 | Glycosyl Hydrolases Family 6 |
|  |  | 2600294816 | Pts System, Glucose Subfamily, Iia Component |
|  |  | 2600295146 | Trehalose-6-Phosphate Synthase |
|  |  | 2600295710 | Udp-Glucose Pyrophosphorylase |
|  |  | 2600295965 | Alpha-1,4-Glucan:Alpha-1,4-Glucan 6-Glycosyltransferase |
|  |  | 2600295964 | Alpha-1,4-Glucan:Maltose-1-Phosphate Maltosyltransferase |
|  |  | 2600295963 | Alpha-Glucan Phosphorylases |
|  |  | 2600298586 | Glucose-1-Phosphate Adenylyltransferase |
|  |  | 2600296108 | Glucose-1-Phosphate Cytidylyltransferase |
|  |  | 2600296833 | Glucose-6-Phosphate Isomerase (Ec 5.3.1.9) |
|  |  | 2600298196 | Glycogen Debranching Enzyme Glgx |
|  |  | 2600298587 | Glycogen Synthase (Adp-Glucose) |
|  |  | 2600297271 | Glycogen/Starch/Alpha-Glucan Phosphorylases |
|  |  | 2600299636 | Haloacid Dehalogenase Superfamily, Subfamily Ia, Variant 3 With Third Motif Having Dd Or Ed |
|  |  | 2600297975 | Malto-Oligosyltrehalose Synthase |
|  |  | 2600298019 | Maltokinase |
|  |  | 2600297976 | Maltooligosyl Trehalose Hydrolase (Ec 3.2.1.141) |
|  |  | 2600299170 | Nucleotide Sugar Dehydrogenase |
|  |  | 2600299293 | Phosphoglucomutase, Alpha-D-Glucose Phosphate-Specific |
|  |  | 2600295073 | Trehalose 6-Phosphatase (Ec 3.1.3.12) |
|  |  | 2600298020 | Trehalose Synthase |
|  |  | 2600295832 | Trehalose-Phosphatase |
|  |  | | |
| **G15** | **Glycolysis/ Gluconeogenesis** | 2600294762 | 6-Phosphofructokinase (Ec 2.7.1.11) |
|  |  | 2600296263 | Acyl-Coenzyme A Synthetases/Amp-(Fatty) Acid Ligases |
|  |  | 2600296411 | Acyl-Coenzyme A Synthetases/Amp-(Fatty) Acid Ligases |
|  |  | 2600298319 | Alcohol Dehydrogenase Groes-Like Domain/Zinc-Binding Dehydrogenase |
|  |  | 2600298990 | Fructose-2,6-Bisphosphatase |
|  |  | 2600295028 | Nad-Dependent Aldehyde Dehydrogenases |
|  |  | 2600296994 | Nad-Dependent Aldehyde Dehydrogenases |
|  |  | 2600298807 | Nad-Dependent Aldehyde Dehydrogenases |
|  |  | 2600295095 | Ndma-Dependent Alcohol Dehydrogenase, Rxyl_3153 Family |
|  |  | 2600295174 | Ndma-Dependent Alcohol Dehydrogenase, Rxyl_3153 Family |
|  |  | 2600299105 | Ndma-Dependent Alcohol Dehydrogenase, Rxyl_3153 Family |
|  |  | 2600294816 | Pts System, Glucose Subfamily, Iia Component |
|  |  | 2600299196 | Phosphoenolpyruvate Carboxykinase (Gtp) |
|  |  | 2600296265 | Pyruvate/2-Oxoglutarate Dehydrogenase Complex, Dehydrogenase (E1) Component, Eukaryotic Type, Alpha Subunit |
|  |  | 2600296594 | Pyruvate/2-Oxoglutarate Dehydrogenase Complex, Dehydrogenase (E1) Component, Eukaryotic Type, Beta Subunit |
|  |  | 2600296595 | Pyruvate/2-Oxoglutarate Dehydrogenase Complex, Dihydrolipoamide Acyltransferase (E2) Component, And Related Enzymes |
|  |  | 2600298350 | Pyruvate/2-Oxoglutarate Dehydrogenase Complex, Dihydrolipoamide Acyltransferase (E2) Component, And Related Enzymes |
|  |  | 2600299600 | Pyruvate/2-Oxoglutarate Dehydrogenase Complex, Dihydrolipoamide Dehydrogenase (E3) Component, And Related Enzymes |
|  |  | 2600299081 | Transcriptional Regulator/Sugar Kinase |
|  |  | 2600298187 | Zn-Dependent Alcohol Dehydrogenases |
|  |  | 2600298320 | Acetaldehyde Dehydrogenase (Ec 1.2.1.10)/Alcohol Dehydrogenase Adhe (Ec 1.1.1.1) |
|  |  | 2600294871 | Acetyl-Coenzyme A Synthetase (Ec 6.2.1.1) |
|  |  | 2600295181 | Aldehyde Dehydrogenase, Rv0768 Family |
|  |  | 2600296704 | Aldehyde Dehydrogenase, Rv0768 Family |
|  |  | 2600298740 | Dihydrolipoamide Dehydrogenase |
|  |  | 2600296743 | Enolase (EC 4.2.1.11) |
|  |  | 2600298869 | Fructose-1,6-Bisphosphatase, Class Ii |
|  |  | 2600296229 | Fructose-Bisphosphate Aldolase (Ec 4.1.2.13) |
|  |  | 2600299441 | Fructose-Bisphosphate Aldolase (Ec 4.1.2.13) |
|  |  | 2600296833 | Glucose-6-Phosphate Isomerase (Ec 5.3.1.9) |
|  |  | 2600299152 | Glucosyl-3-Phosphoglycerate Phosphatase (Pgm Family) |
|  |  | 2600297226 | Glyceraldehyde-3-Phosphate Dehydrogenase, Type I |
|  |  | 2600295827 | Hexose Kinase, 1-Phosphofructokinase Family |
|  |  | 2600298670 | Malate Dehydrogenase (Nad) (Ec 1.1.1.37) |
|  |  | 2600299293 | Phosphoglucomutase, Alpha-D-Glucose Phosphate-Specific |
|  |  | 2600298768 | Phosphoglycerate Mutase (Ec 5.4.2.1) |
|  |  | 2600296593 | Pyruvate Dehydrogenase E1 Component, Alpha Subunit |
|  |  | 2600297252 | Pyruvate Dehydrogenase E1 Component, Homodimeric Type |
|  |  | 2600297934 | Pyruvate Kinase (Ec 2.7.1.40) |
|  |  | | |
| **G15** | **Pentose phosphate pathway** | 2600294762 | 6-Phosphofructokinase (Ec 2.7.1.11) |
|  |  | 2600298702 | 6-Phosphogluconate Dehydrogenase (Decarboxylating) |
|  |  | 2600298127 | 6-Phosphogluconate Dehydrogenase (Decarboxylating) (Ec 1.1.1.44) |
|  |  | 2600299159 | Sugar Kinases, Ribokinase Family |
|  |  | 2600298756 | Deoxyribose-Phosphate Aldolase |
|  |  | 2600298869 | Fructose-1,6-Bisphosphatase, Class Ii |
|  |  | 2600296229 | Fructose-Bisphosphate Aldolase (Ec 4.1.2.13) |
|  |  | 2600299441 | Fructose-Bisphosphate Aldolase (Ec 4.1.2.13) |
|  |  | 2600299550 | Gluconate Kinase, Ski Family (Ec 2.7.1.12) |
|  |  | 2600296018 | Glucose-6-Phosphate 1-Dehydrogenase (Ec 1.1.1.49) |
|  |  | 2600297211 | Glucose-6-Phosphate 1-Dehydrogenase (Ec 1.1.1.49) |
|  |  | 2600296833 | Glucose-6-Phosphate Isomerase (Ec 5.3.1.9) |
|  |  | 2600295827 | Hexose Kinase, 1-Phosphofructokinase Family |
|  |  | 2600299293 | Phosphoglucomutase, Alpha-D-Glucose Phosphate-Specific |
|  |  | 2600296633 | Ribose 5-Phosphate Isomerase |
|  |  | 2600296732 | Ribose-Phosphate Pyrophosphokinase |
|  |  | 2600297210 | Transaldolase (EC 2.2.1.2) |
| **G21: Unclutured *Smithella* Species** | | | |
| **G21** | **Hydrogen production** | 2603688800 | Nadh:Ubiquinone Oxidoreductase, Nadh-Binding (51 Kd) Subunit |
|  |  | 2603688801 | Ferredoxin |
|  |  | 2603688802 | Nad(P)-Dependent Iron-Only Hydrogenase Diaphorase Component Iron-Sulfur Protein |
|  | **Formate oxidation** | 2603687950 | Formate Dehydrogenase Family Accessory Protein Fdhd |
|  |  | 2603688077 | Formate Dehydrogenase Family Accessory Protein Fdhd |
|  |  | 2603688919 | Uncharacterized Protein Involved In Formate Dehydrogenase Formation |
|  |  | 2603688953 | Cytochrome B Subunit Of Formate Dehydrogenase |
|  |  | 2603689899 | Formate Dehydrogenase (Quinone-Dependent) Iron-Sulfur Subunit |
|  |  | 2603689900 | Formate Dehydrogenase, Gamma Subunit |
|  |  | 2603690023 | Uncharacterized Protein Involved In Formate Dehydrogenase Formation |
|  |  | 2603690025 | Formate Dehydrogenase (Nadp) Alpha Subunit (Ec 1.2.1.43) |
|  |  | 2603690027 | Formate Dehydrogenase, Alpha Subunit, Archaeal-Type |
|  |  | | |
| **G21** | **Sulfite reduction** | 2603687864 | Dissimilatory Sulfite Reductase Alpha Subunit (Dsra; EC 1.8.99.1) |
|  |  | 2603687865 | Dissimilatory Sulfite Reductase Beta Subunit (Dsra; EC 1.8.99.1) |
|  |  | 2603687866 | Dissimilatory Sulfite Reductase D (Dsrd) |
|  |  | 2603687874 | Putative Sulfite Reductase-Associated Electron Transfer Protein Dsrk |
|  |  | 2603687875 | Putative Sulfite Reductase-Associated Electron Transfer Protein Dsrj |
|  |  | 2603687876 | Putative Sulfite Reductase-Associated Electron Transfer Protein Dsro |
|  | **Polysufide reduction** | 2603687877 | Polysulphide Reductase, Nrfd |
|  |  | 2603687878 | Polysulphide Reductase, Nrfd |
|  |  | 2603687941 | Polysulphide Reductase |
|  |  | | |
| **G21** | **Alcohol dehydrogenation** | 2603687637 | Alcohol Dehydrogenase, Class Iv |
|  |  | 2603687638 | Alcohol Dehydrogenase, Class Iv |
|  |  | 2603687639 | Aldehyde:Ferredoxin Oxidoreductase |
|  |  | 2603687646 | Alcohol Dehydrogenase, Class Iv |
|  |  | 2603687706 | Dehydrogenases Related To Short-Chain Alcohol Dehydrogenases |
|  |  | 2603687728 | Dehydrogenases Related To Short-Chain Alcohol Dehydrogenases |
|  |  | 2603687745 | Dehydrogenases Related To Short-Chain Alcohol Dehydrogenases |
|  |  | 2603687882 | Dehydrogenases Related To Short-Chain Alcohol Dehydrogenases |
|  |  | 2603687907 | Alcohol Dehydrogenase Groes-Like Domain |
|  |  | 2603687926 | Zn-Dependent Alcohol Dehydrogenases |
|  |  | 2603688257 | Alcohol Dehydrogenase, Class Iv |
|  |  | 2603688484 | Iron-Containing Alcohol Dehydrogenase |
|  |  | 2603688485 | Iron-Containing Alcohol Dehydrogenase |
|  |  | 2603688555 | Short-Chain Alcohol Dehydrogenase Of Unknown Specificity |
|  |  | 2603688999 | Alcohol Dehydrogenase, Class Iv |
|  |  | 2603689000 | Nad-Dependent Aldehyde Dehydrogenases |
|  |  | 2603689001 | Aldehyde:Ferredoxin Oxidoreductase |
|  |  | 2603689935 | Alcohol Dehydrogenase Groes-Like Domain |
|  |  | | |
| **G21** | **Propionate degradation: (I) MMC pathway and (II) Acryloyl-CoA pathway** | 2603687952 | Methylmalonyl-Coa Epimerase (Ec 5.1.99.1) |
|  |  | 2603687963 | Methylmalonyl-Coa Mutase (Ec 5.4.99.2) |
|  |  | 2603689026 | Methylmalonyl-Coa Mutase (Ec 5.4.99.2) |
|  |  | 2603689027 | Methylmalonyl-Coa Mutase C-Terminal Domain |
|  |  | 2603688505 | Propionate Coa-Transferase (Ec 2.8.3.1) |
|  |  | 2603688855 | Propionate Coa-Transferase (Ec 2.8.3.1) |
|  |  | 2603688286 | 2-Oxoacid:Acceptor Oxidoreductase, Delta Subunit |
|  |  | 2603688300 | 2-Oxoacid:Acceptor Oxidoreductase, Delta Subunit |
|  |  | 2603688301 | 2-Oxoacid:Acceptor Oxidoreductase, Gamma Subunit |
|  |  | 2603689357 | Acetate Kinase |
|  |  | 2603688687 | Acetoacetate Decarboxylase |
|  |  | 2603688278 | Acetyl-Coa Acetyltransferases |
|  |  | 2603688565 | Acetyl-Coa Acetyltransferases |
|  |  | 2603689916 | Acetyl-Coenzyme A Synthetase (Ec 6.2.1.1) |
|  |  | 2603688137 | Acyl-Coa Dehydrogenases |
|  |  | 2603688208 | Acyl-Coa Dehydrogenases |
|  |  | 2603689771 | Acyl-Coa Synthetase (Ndp Forming) |
|  |  | 2603689543 | Biotin-Requiring Enzyme |
|  |  | 2603688143 | Enoyl-Coa Hydratase/Carnithine Racemase |
|  |  | 2603688145 | Enoyl-Coa Hydratase/Carnithine Racemase |
|  |  | 2603689009 | Glycerol Dehydratase, Cobalamin-Independent, Large Subunit (Ec 4.2.1.30) |
|  |  | 2603689000 | Nad-Dependent Aldehyde Dehydrogenases |
|  |  | 2603689406 | Nad-Dependent Aldehyde Dehydrogenases |
|  |  | 2603688299 | Pyruvate Ferredoxin Oxidoreductase, Alpha Subunit (Ec 1.2.7.1) |
|  |  | 2603688298 | Pyruvate Ferredoxin Oxidoreductase, Beta Subunit (Ec 1.2.7.1) |
|  |  | 2603688285 | Pyruvate Ferredoxin Oxidoreductase, Gamma Subunit (Ec 1.2.7.1) |
|  |  | 2603688287 | Pyruvate:Ferredoxin Oxidoreductase, Alpha Subunit |
|  |  | 2603688288 | Pyruvate:Ferredoxin Oxidoreductase, Beta Subunit |
|  |  | 2603689827 | Pyruvate-Formate Lyase |
|  |  | | |
| **G21** | **Butyrate degradation**  **(via crotonoyl-CoA to** **acetyl-CoA)** | 2603687619 | Acyl Coa:Acetate Coa Transferase, Alpha Subunit |
|  |  | 2603687620 | Acyl Coa:Acetate Coa Transferase, Beta Subunit |
|  |  | 2603687768 | Acyl-Coa Synthetases (AMP-Forming)/AMP-Acid Ligases II |
|  |  | 2603687769 | Acyl-Coa Synthetases (AMP-Forming)/AMP-Acid Ligases II |
|  |  | 2603688566 | Butyryl-Coa Dehydrogenase |
|  |  | 2603689005 | Butyryl-Coa Dehydrogenase |
|  |  | 2603688143 | Enoyl-Coa Hydratase/Carnithine Racemase |
|  |  | 2603688423 | Enoyl-Coa Hydratase/Carnithine Racemase |
|  |  | 2603689483 | Enoyl-Coa Hydratase/Carnithine Racemase |
|  |  | 2603687614 | Acetyl-Coa C-Acetyltransferase |
|  |  | 2603687615 | Acetyl-Coa C-Acetyltransferase |
|  |  | 2603687622 | Acetyl-Coa C-Acetyltransferase |
|  |  | 2603687623 | Acetyl-Coa C-Acetyltransferase |
|  |  | 2603688409 | 3-Hydroxybutyryl-Coa Dehydrogenase |
|  |  | 2603689529 | 3-Hydroxybutyryl-Coa Dehydrogenase |
|  |  | 2603688896 | 3-Hydroxyacyl-Coa Dehydrogenase, NAD Binding Domain |
|  |  | 2603688897 | 3-Hydroxyacyl-Coa Dehydrogenase, C-Terminal Domain |
|  |  | 2603688023 | 3-Hydroxybutyryl-Coa Dehydratase |
